# De novo profiling of RNA viruses in *Anopheles* malaria vector mosquitoes from forest ecological zones in Senegal and Cambodia

**DOI:** 10.1186/s12864-019-6034-1

**Published:** 2019-08-20

**Authors:** Eugeni Belda, Ferdinand Nanfack-Minkeu, Karin Eiglmeier, Guillaume Carissimo, Inge Holm, Mawlouth Diallo, Diawo Diallo, Amélie Vantaux, Saorin Kim, Igor V. Sharakhov, Kenneth D. Vernick

**Affiliations:** 1 Unit of Insect Vector Genetics and Genomics, Department of Parasites and Insect Vectors, Institut Pasteur, Paris, France; 2 CNRS Unit of Evolutionary Genomics, Modeling, and Health (UMR2000), Institut Pasteur, Paris, France; 3 Integromics Unit, Institute of Cardiometabolism and Nutrition, Assistance Publique Hôpitaux de Paris, Pitié-Salpêtrière Hospital, Paris, France; 4 Sorbonne Université, Graduate School of Life Sciences ED515, UPMC - Université Pierre et Marie Curie - Paris 6, 4 Place Jussieu, 75252 Paris, France; 5 Laboratory of Microbial Immunity, Singapore Immunology Network, Agency for Science, Technology and Research (A(∗)STAR), Singapore, Singapore; 6 Institut Pasteur de Dakar, Dakar, Senegal; 7 Institut Pasteur of Cambodia, Phnom Penh, Cambodia; 8 Department of Entomology, Virginia Polytechnic Institute and State University, Blacksburg, VA USA

**Keywords:** Virus genome assembly, Insect specific virus, RNA virus, *Anopheles*, Malaria vector, Virome

## Abstract

**Background:**

Mosquitoes are colonized by a large but mostly uncharacterized natural virome of RNA viruses, and the composition and distribution of the natural RNA virome may influence the biology and immunity of *Anopheles* malaria vector populations.

**Results:**

*Anopheles* mosquitoes were sampled in malaria endemic forest village sites in Senegal and Cambodia, including *Anopheles funestus, Anopheles gambiae* group sp., and *Anopheles coustani* in Senegal, and *Anopheles hyrcanus* group *sp., Anopheles maculatus* group *sp*.*,* and *Anopheles dirus* in Cambodia. The most frequent mosquito species sampled at both study sites are human malaria vectors. Small and long RNA sequences were depleted of mosquito host sequences, de novo assembled and clustered to yield non-redundant contigs longer than 500 nucleotides. Analysis of the assemblies by sequence similarity to known virus families yielded 115 novel virus sequences, and evidence supports a functional status for at least 86 of the novel viral contigs. Important monophyletic virus clades in the *Bunyavirales* and *Mononegavirales* orders were found in these *Anopheles* from Africa and Asia. The remaining non-host RNA assemblies that were unclassified by sequence similarity to known viruses were clustered by small RNA profiles, and 39 high-quality independent contigs strongly matched a pattern of classic RNAi processing of viral replication intermediates, suggesting they are entirely undescribed viruses. One thousand five hundred sixty-six additional high-quality unclassified contigs matched a pattern consistent with Piwi-interacting RNAs (piRNAs), suggesting that strand-biased piRNAs are generated from the natural virome in *Anopheles*. To functionally query piRNA effect, we analyzed piRNA expression in *Anopheles coluzzii* after infection with O’nyong nyong virus (family *Togaviridae*), and identified two piRNAs that appear to display specifically altered abundance upon arbovirus infection.

**Conclusions:**

*Anopheles* vectors of human malaria in Africa and Asia are ubiquitously colonized by RNA viruses, some of which are monophyletic but clearly diverged from other arthropod viruses. The interplay between small RNA pathways, immunity, and the virome may represent part of the homeostatic mechanism maintaining virome members in a commensal or nonpathogenic state, and could potentially influence vector competence.

**Electronic supplementary material:**

The online version of this article (10.1186/s12864-019-6034-1) contains supplementary material, which is available to authorized users.

## Background

*Anopheles* mosquitoes are the only vectors of human malaria, which kills at least 400,000 persons and causes 200 million cases per year, with the greatest impact concentrated in sub-Saharan Africa and South-East Asia [1]. In addition to malaria, *Anopheles* mosquitoes also transmit the alphavirus O’nyong nyong (ONNV, family *Togaviridae*), which is the only arbovirus known to employ *Anopheles* mosquitoes as the primary vector [2, 3]. A recent review found reports of at least 51 viruses naturally associated with *Anopheles* [2], and *Anopheles* mosquitoes harbor a diverse natural virome of RNA viruses [4–7]. These viruses are comprised mainly of insect specific viruses (ISVs) thought to multiply only in insects, but also includes relatives of arboviruses that can replicate in both insects and vertebrate cells.

*Anopheles* viruses have been discovered by isolation from cultured cells exposed to mosquito extract, serology, specific amplification and sequencing, and more recently, deep sequencing and de novo assembly [2]. Although this work has increased the number of ISVs discovered in *Anopheles*, there are probably many still unknown. Because *Anopheles* mosquitoes are not thought to be important arbovirus vectors, relatively little research has been done on their viruses. In contrast, culicine mosquitoes in the genera *Aedes* and *Culex* transmit multiple arboviruses such as dengue virus (DENV, family *Flaviviridae*) Zika virus (ZIKV, family *Flaviviridae*), chikungunya virus (CHIKV, family *Togaviridae*) and others, but do not transmit human malaria.

Here, we assembled small and long RNA sequences from wild *Anopheles* mosquitoes captured in forest ecologies in central and northern Cambodia and eastern Senegal. The collection sites are considered disease emergence zones, with high levels of fevers and encephalopathies of unknown origin.

It is important to study *Anopheles* viruses because persistent exposure to ISVs, rather than the relatively infrequent exposure to arboviruses such as ONNV, has probably been the main evolutionary pressure shaping *Anopheles* antiviral immunity. *Anopheles* resistance mechanisms against arbovirus infection may be particularly efficient, given their low level of known arbovirus transmission despite highly anthropophilic feeding behavior, including on viremic hosts. Nevertheless, ONNV transmission indicates that arbovirus transmission by *Anopheles* is at least possible, so it is worth asking why transmission by *Anopheles* is apparently restricted to a single known virus. Identifying the complement of natural viruses inhabiting the *Anopheles* niche will help clarify the biology underlying the apparent inefficiency of arbovirus transmission by *Anopheles*, and may suggest new translational tools to decrease arbovirus transmission by the more efficient *Aedes* and *Culex* vectors.

## Results

### Mosquito species estimation

**Fig. 1:**
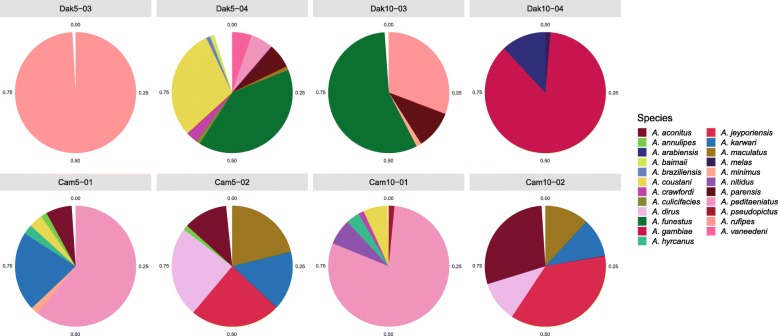
Taxonomic profile of *Anopheles* sample pools. Relative abundance values of *Anopheles* species were computed by mapping long RNAseq reads to mitochondrial cytochrome C oxidase subunit I gene sequences from the Barcode of Life COI-5P Database. Taxa represented by > 100 sequence reads and 1% frequency in the sample pool were plotted in pie charts. White wedges in pie charts represent the combined proportion of all sequence matches that were individually present at less than 1% frequency in the sample. All data are presented in tabular form in Additional file 1: Table S1

Metagenomic sequencing of long and small fractions of RNA was carried out for four biological replicate pools of mosquitoes from Ratanakiri and Kampong Chhnang provinces in central and northern Cambodia near the border with Laos, and four replicate pools from Kedougou in eastern Senegal near the border with the Republic of Guinea (Conakry). Mosquito species composition of sample pools was estimated using sequences of transcripts from the mitochondrial cytochrome c oxidase subunit 1 (COI) gene, which were compared with *Anopheles* sequences from the Barcode of Life COI-5P database (Fig. 1, Additional file 1: Table S1). In the Senegal samples, the most frequent mosquito species were *Anopheles rufipes*, *Anopheles funestus, Anopheles gambiae* group sp., and *Anopheles coustani*, which are all human malaria vectors, including the recently incriminated *An. rufipes* [8]. In the Cambodia samples, the most frequent species were *Anopheles hyrcanus* group *sp., Anopheles maculatus* group *sp*.*, Anopheles karwari, Anopheles jeyporeisis, Anopheles aconitus* and *Anopheles dirus*. All are considered human malaria vectors [9–12]. Elevated rates of human blood-feeding by a mosquito species is a prerequisite for malaria vectorial capacity [13], and therefore the main *Anopheles* species sampled for virome discovery in this study display consistently high levels of human contact in nature. In addition, a number of rare mosquito species calls represent species supported by less than 100 sequence reads and 1% frequency in the sample pool (Additional file 1: Table S1). These rare calls could result from sequencing technical artifacts, mutations of COI, errors in the COI-5P database, and/or undescribed mosquito taxa not in the database.

### Virus discovery by de novo assembly and classification by sequence similarity

Small and long RNAseq reads were de novo assembled after removal of mosquito sequences. Non-redundant contigs longer than 500 nucleotides from assemblies of both sampling sites, Cambodia and Senegal, were translated into predicted peptide sequences and used to search the GenBank protein sequence database using BLASTX with an e-value threshold of 1e-10. BLASTX translates a DNA sequence in all six putative reading frames and searches against a protein database to identify peptide homology matches. This analysis pipeline allowed identification of 115 novel assembled virus sequences, 37 from the Senegal samples (virus ID suffix “Dak”, Table 1), and 78 from the Cambodia samples (virus ID suffix “Camb”, Table 2), possibly pointing to higher viral diversity in mosquitoes from Cambodia. Some of the 115 virus sequences showed remote similarity by BLASTX to 24 reference viruses in GenBank that include single-stranded RNA (ssRNA)-negative strand viruses of the families *Orthomyxoviridae*, *Rhabdoviridae* and *Phenuiviridae,* ssRNA positive-strand viruses of the families *Virgaviridae*, *Flaviviridae* and *Bromoviridae*, dsRNA viruses of the family *Reoviridae* and multiple unclassified viruses of both ssRNA and dsRNA types (Table 3). Most of these remote similarities were with viruses characterized in a recent virus survey of 70 different arthropod species collected in China [14], which emphasizes the importance of high throughput surveys of the arthropod virosphere in the identification of viruses associated with different arthropod species.

**Table 1:** Summary of virus assemblies, Senegal *Anopheles* sample pools

Reference virus	3NCBI classification reference virus	Closest relative (BLASTX)	Assembled sequence	Alignment length (identities, positives, gaps, frame)	Score (E-value) Blastx	Alignment coordinates (start..end Assembled sequence-start..end Closest relative)
DsRNA virus environmental sample clone mill.culi_contig84	Viruses; dsRNA viruses; environmental samples.	gi|766989332|gb|AJT39580.1| proline-alanine-rich protein [dsRNA virus environmental sample] (975 aa)	PrAlaRichProt_EnvVirDak (1345 nt)	308 (71,122,6,+ 1)	77(1e-11)	4..909–653..954
Homalodisca vitripennis reovirus segment S3	Viruses; dsRNA viruses; Reoviridae; Sedoreovirinae; Phytoreovirus; unclassified Phytoreovirus	gi|226423326|ref.|YP_002790886.1| major core protein [Homalodisca vitripennis reovirus] (1021 aa)	CP_HVreovirusDak (5674 nt)	483 (113,206,21,+ 3)	90(2e-14)	3774..5159–211..671
Daeseongdong virus 1 strain A12.2708/ROK/2012	Viruses; unclassified viruses.	gi|959121745|ref.|YP_009182191.1| putative RNA-dependent RNA polymerase [Daeseongdong virus 1]. (2361 aa)	RdRP_DaeseondongVirDak (2530 nt)	503 (125,225,6,-1)	135(1e-28)	875..2365–45..489
Ixodes scapularis associated virus 2 isolate A1, partial genome	Viruses; unclassified viruses.	gi|669132782|gb|AII01812.1| hypothetical protein, partial [Ixodes scapularis associated virus 2] (337 aa)	HP1.1_IxodesVirDak (2820 nt)	340 (141,210,2,-2)	267(3e-77)	105..1118–3..337
			HP1.2_IxodesVirDak (2561 nt)	319 (116,157,11,-1)	159(4e-39)	156..1079–3..319
Uncultured virus isolate acc_7.4	Viruses; environmental samples.	gi|545716017|gb|AGW51759.1| RNA-dependent RNA polymerase-like protein [uncultured virus] (473 aa)	RdRP_UncVir1Dak (1488 nt)	475 (217,295,8,-2)	377(e-121)	57..1457–1..471
Uncultured virus isolate acc_1.3	Viruses; environmental samples.	gi|545716010|gb|AGW51755.1| RNA-dependent RNA polymerase-like protein, partial [uncultured virus] (1445 aa)	RdRP_UncVir2Dak (2011 nt)	236 (168,200,0,+ 1) 178 (146,157,0,+ 3) 91 (71,80,0,+ 2)	353 (0.0) 298 (0.0) 152 (0.0)	25..732–58..293 1020..1553–408..585 752..1024–320..410
American dog tick phlebovirus isolate FI3	Viruses; ssRNA viruses; ssRNA negative-strand viruses; Bunyavirales; Phlebovirus; unclassified Phlebovirus.	gi|734669629|gb|AJA31764.1| nucleocapsid, partial [American dog tick phlebovirus] (245 aa)	NuclCap1.1_ADTphlebovirusDak (1105 nt)	142 (55,75,11,+ 2)	75(5e-12)	191..583–38..175
			NuclCap1.2_ADTphlebovirusDak (1148 nt)	151 (53,71,9,+ 2)	72(3e-11)	173..598–27..175
Culex tritaeniorhynchus rhabdovirus RNA, complete genome, strain: TY	Viruses; ssRNA viruses; ssRNA negative-strand viruses; Mononegavirales; Rhabdoviridae; unclassified Rhabdoviridae.	gi|700895640|ref.|YP_009094323.1| large protein [Culex tritaeniorhynchus rhabdovirus] (2123 aa)	LP_CulexRhabdovDak (1526 nt)	266 (101,149,0,+ 3) 108 (33,45,0,+ 2) 72 (50,59,0,+ 2)	177(6e-52) 56(6e-52) 115(2e-23)	498..1295–155..417 149..472–38..145 1307..1522–593..664
Phasi Charoen-like virus	Viruses; ssRNA viruses; ssRNA negative-strand viruses; Bunyaviridae; unclassified Bunyaviridae.	gi|664682120|gb|AIF71032.1| nucleocapsid [Phasi Charoen-like virus] (268 aa)	NuclCap1.2_PCLVDak (1104 nt)	82 (49,57,1,+ 3)	84(6e-26)	699..941–116..197
			NuclCap1.3_PCLVDak (1125 nt)	97 (57,74,0,+ 2)	115(7e-48)	119..409–65..161
			NuclCap1.4_PCLVDak (1144 nt)	262 (114,167,3,+ 3)	217(7e-64)	153..929–6..267
		gi|870898373|gb|AKP18600.1| glycoprotein [Phasi Charoen-like virus] (1237 aa)	GP_PCLVDak (3887 nt)	1185 (436,711,15,+ 1)	894 (0.0)	91..3600–20..1197
		gi|664682116|gb|AIF71030.1| RNA-dependent RNA polymerase [Phasi Charoen-like virus] (2217 aa)	RdRP_PCLVDak (6711 nt)	2213 (1042,1432,52,+ 1)	1944 (0.0)	148..6630–8..2196
Wellfleet Bay virus isolate 10–280-G segment 4	Viruses; ssRNA viruses; ssRNA negative-strand viruses; Orthomyxoviridae; Quaranjavirus; unclassified Quaranjavirus.	gi|727361119|ref.|YP_009110683.1| nucleoprotein [Wellfleet Bay virus] (537 aa)	Nuclprot1.1_WBvirDak (1973 nt)	213 (60,101,7,+ 3)	68(2e-08)	705..1322–149..354
			Nuclprot1.2_WBvirDak (3252 nt)	459 (114,197,39,+ 3)	117(8e-24)	1413..2672–11..451
Wuhan Mosquito Virus 9 strain JX1–13	Viruses; ssRNA viruses; ssRNA negative-strand viruses; unclassified ssRNA negative-strand viruses.	gi|752455731|gb|AJG39214.1| ORF1 [Wuhan Mosquito Virus 9] (279 aa)	ORF1_Wuhan9virDak (1574 nt)	204 (57,94,2,+ 3)	86(3e-15)	867..1472–13..210
Wuhan Mosquito Virus 1 strain WT3–15	Viruses; ssRNA viruses; ssRNA negative-strand viruses; unclassified ssRNA negative-strand viruses.	gi|752455880|gb|AJG39296.1| glycoprotein precursor [Wuhan Mosquito Virus 1]. (681 aa)	GP_Wuhan1virDak (3547 nt)	153 (97,122,11,+ 1)	219(3e-56)	2023..2448–243..395
Wuhan Spider Virus strain SYZZ-2	Viruses; ssRNA viruses; ssRNA negative-strand viruses; unclassified ssRNA negative-strand viruses.	gi|752455826|gb|AJG39269.1| RNA-dependent RNA polymerase [Wuhan Spider Virus] (2251 aa)	RdRP1.1_WSVDak (998 nt)	354 (87,154,26,+ 3)	104(1e-20)	6..989–1239..1589
			RdRP1.2_WSVDak (2083 nt)	730 (209,327,36 + 2)	263(1e-70)	2..2083–531..1243
			RdRP1.3_WSVDak (1070 nt)	377 (108,172,23 + 2)	139(9e-32)	2..1063–579..951
Xincheng Mosquito Virus strain XC1–6	Viruses; ssRNA viruses; ssRNA negative-strand viruses; unclassified ssRNA negative-strand viruses.	gi|752455743|gb|AJG39224.1| ORF1 [Xincheng Mosquito Virus] (462 aa)	ORF1_XinchengVirDak (947 nt)	299 (73,122,11,+ 2)	55(5e-05)	81..944–22..311
		gi|752455744|gb|AJG39225.1| ORF2 [Xincheng Mosquito Virus] (437 aa)	ORF2_XinchengVirDak (1726 nt)	165 (44,88,2,+ 1)	67(2e-08)	145..633–260..422
		gi|752455745|gb|AJG39226.1| glycoprotein [Xincheng Mosquito Virus] (648 aa)	GP_XinchengVirDak (5993 nt)	592 (352,445,0,+ 3)	751 (0.0)	3720..5495–16..607
		gi|752455746|gb|AJG39227.1| RNA-dependent RNA polymerase [Xincheng Mosquito Virus] (2026 aa)	RdRP1.1_XinchengVirDak (11707 nt)	2001 (733,1101,50,+ 3)	1226 (0.0)	5571..11423–28..1977
			RdRP1.2_XinchengVirDak (11722 nt)	2001 (733,1101,50,+ 3)	1226 (0.0)	5586..11438–28..1977
			RdRP1.3_XinchengVirDak (11710 nt)	2001 (734,1104,50,+ 3)	1229 (0.0)	293..6145–28..1977
			RdRP1.4_XinchengVirDak (11694 nt)	2001 (734,1104,50,-3)	1229 (0.0)	293..6145–28..1977
			RdRP1.5_XinchengVirDak (11728 nt)	1955 (733,1093,47,-1)	1233 (0.0)	440..6163–28..1924
			RdRP1.6_XinchengVirDak (11716 nt)	1955 (733,1093,47,+ 2)	1233 (0.0)	5561..11284–28..1924
			RdRP1.7_XinchengVirDak (6128 nt)	1962 (1088,1397,9,-3)	2179 (0.0)	124..5982–36..1994
Xinzhou Mosquito Virus strain XC3–5	Viruses; ssRNA viruses; ssRNA negative-strand viruses; unclassified ssRNA negative-strand viruses.	gi|752455830|gb|AJG39271.1| RNA-dependent RNA polymerase [Xinzhou Mosquito Virus]. (2022 aa)	RdRP1.1_XinzhouVirDak (7527 nt)	2017 (1403,1670,3,+ 1)	2863 (0.0)	1315..7356–1..2013
			RdRP1.2_XinzhouVirDak (7524 nt)	2020 (1403,1676,6,+ 1)	2864 (0.0)	1312..7353–1..2013
Sunn-hemp mosaic virus	Viruses; ssRNA viruses; ssRNA positive-strand viruses, no DNA stage; Virgaviridae; Tobamovirus.	gi|12643499|sp.|P89202.2|RDRP_SHMV RecName: Full = Replicase large subunit] (1629 aa)	RdRP_SHMVDak (1216 nt)	365 (105,171,13,-1)	125(4e-27)	2..1057–932..1240
Omono River virus	Viruses; dsRNA viruses	gi|307933351|dbj|BAJ21511.1| RNA-dependent RNA polymerase [Omono River virus] (621 aa)	RdRP_OmonoVirDak (613 nt)	194 (71,110,1,-1)	131(1e-31)	5..583–135..323
Beaumont virus strain 6	Viruses; ssRNA viruses; ssRNA negative-strand viruses; Mononegavirales; Rhabdoviridae; unclassified Rhabdoviridae.	gi|550631504|gb|AGX86091.1| RNA-dependent RNA polymerase, partial [Beaumont virus] (1568 aa)	RdRP_BeaumontVirDak (805 nt)	153 (76,98,0,+ 3)	142(9e-34)	3..461–417..569

**Table 2:** Summary of virus assemblies, Cambodia *Anopheles* sample pools

Reference virus	NCBI classification reference virus	Closest relative (BLASTX)	Assembled sequence	Alignment length (identities, positives, gaps, frame)	Score (E-value) Blastx	Alignment coordinates (start..end Assembled sequence-start..end Closest relative)
uncultured virus	Viruses; environmental samples.	RNA-dependent RNA polymerase-like protein, partial [uncultured virus] (KF298266.1) (1445 aa)	RdRP1.1_UncVir2Camb (857 nt)	261 (176,212,0,+ 3)	379(e-117)	75..857–1..261
			RdRP1.2_UncVir2Camb (797 nt)	194 (135,161,0,+ 2)	282(8e-83)	212..793–102..295
			RdRP1.3_UncVir2Camb (2177 nt)	486 (350,408,0,+ 1)	731 (0.0)	718..2175–1..486
			RdRP1.4_UncVir2Camb (2574 nt)	748 (525,619,0,+ 3)	1063 (0.0)	6..2249–567..1314
			RdRP1.6_UncVir2Camb (1716 nt)	365 (274,312,0,+ 3) 83 (61,72,0,+ 1)	563 (0.0) 132 (0.0)	315..1409–85..449 64..312–1..83
Culex tritaeniorhynchus rhabdovirus RNA, complete genome; NC_025384	Viruses; ssRNA viruses; ssRNA negative-strand viruses; Mononegavirales; Rhabdoviridae; unclassified Rhabdoviridae.	gi|700895639|ref.|YP_009094322.1| glycoprotein [Culex tritaeniorhynchus rhabdovirus] (503 aa)	GP_CulexRhabdovCamb (2866 nt)	360 (85,130,15,+ 1)	83(6e-13)	1261..2295–49..397
		gi|700895640|ref.|YP_009094323.1| large protein [Culex tritaeniorhynchus rhabdovirus] (2123 aa)	LP1.1_CulexRhabdovCamb (755 nt)	125 (36,58,0,+ 1)	64(2e-08)	64..438–42..166
			LP1.2_CulexRhabdovCamb (1454 nt)	211 (87,130,3,+ 3)	165(1e-39)	498..1121–214..421
Phasi Charoen-like virus	Viruses; ssRNA viruses; ssRNA negative-strand viruses; Bunyavirales; unclassified Bunyavirales	gi|870898373|gb|AKP18600.1| glycoprotein [Phasi Charoen-like virus] (1237 aa)	GP1.1_PCLVCamb (642 nt)	221 (61,110,8,+ 3)	103(2e-21)	3..641–226..444
			GP1.2_PCLVCamb (933 nt)	292 (141,197,0,+ 1)	297(4e-88)	4..879–813..1104
		gi|870898376|gb|AKP18601.1| Nucleocapsid [Phasi Charoen-like virus] (268 aa)	NuclCap1.1_PCLVCamb (1104 nt)	269 (119,165,3,+ 3)	214(4e-63)	207..1004–1..267
			NuclCap1.2_PCLVCamb (533 nt)	113 (48,77,0,+ 2)	97(8e-28)	47..385–3..115
			NuclCap1.3_PCLVCamb (535 nt)	116 (69,90,0,+ 2)	143(4e-38)	119..466–64..179
Bivens Arm virus isolate UF 10	Viruses; ssRNA viruses; ssRNA negative-strand viruses; Mononegavirales; Rhabdoviridae; Tibrovirus	gi|751997168|gb|AJG05818.1| nucleoprotein N [Bivens Arm virus] (428 aa)	NProt_BivArmsVirCamb (516 nt)	162 (47,76,10,+ 1)	52(6e-05)	52..507–22..176
Jurona virus	Viruses; ssRNA viruses; ssRNA negative-strand viruses; Mononegavirales; Rhabdoviridae; Vesiculovirus.	gi|701219310|ref.|YP_009094377.1| polymerase [Jurona virus] > gnl|... 176 2e-43 (2090 aa)	RdRP_JuronaVirCamb (1329 nt)	303 (110,155,0,-2)	176(2e-43)	3..911–1635..1935
Puerto Almendras virus isolate LO-39	Viruses; ssRNA viruses; ssRNA negative-strand viruses; Mononegavirales; Rhabdoviridae; unclassified Rhabdoviridae.	gi|701219331|ref.|YP_009094394.1| L protein [Puerto Almendras vir... 213 2e-54 (2059 aa)	Lprot1.1_PAvirCamb (1869 nt)	587 (163,281,28,-3)	213(2e-54)	191..1867–1461..2039
			Lprot1.2_PAvirCamb (3895 nt)	1308 (657,904,10,-2)	1318 (0.0)	1..3894–161..1467
		gi|701219327|ref.|YP_009094389.1| N protein [Puerto Almendras virus] (434 aa)	Nprot_PAvirCamb (4449 nt)	431 (109,194,18,-2)	123(7e-26)	2964..4202–8..426
Beaumont virus strain 6	Viruses; ssRNA viruses; ssRNA negative-strand viruses; Mononegavirales; Rhabdoviridae; unclassified Rhabdoviridae.	gi|550631504|gb|AGX86091.1| RNA-dependent RNA polymerase, partial [Beaumont virus] (1568 aa)	RdRP1.1_BeaumontVirCamb (586 nt)	81 (42,62,0,+ 2)	100(2e-20)	344..586–187..267
			RdRP1.2_BeaumontVirCamb (633 nt)	187 (106,137,0,+ 2)	233(3e-66)	65..625–1..187
			RdRP1.3_BeaumontVirCamb (594 nt)	156 (98,125,0,+ 1)	198(2e-54)	40..507–1225..1380
			RdRP1.4_BeaumontVirCamb (1359 nt)	452 (288,349,0,+ 3)	592 (0.0)	3..1358–228..679
			RdRP1.5_BeaumontVirCamb (1606 nt)	536 (365,428,1,+ 1)	758 (0.0)	1..1605–26..561
			RdRP1.6_BeaumontVirCamb (1141 nt)	380 (236,287,1,+ 1)	477(e-151)	1..1137–670..1049
			RdRP1.7_BeaumontVirCamb (1667 nt)	324 (169,222,15,+ 3)	317(5e-91)	705..1631–303..622
Wellfleet Bay virus isolate 10–280-G segment 4	Viruses; ssRNA viruses; ssRNA negative-strand viruses; Orthomyxoviridae; Quaranjavirus; unclassified Quaranjavirus.	gi|727361119|ref.|YP_009110683.1| nucleoprotein [Wellfleet Bay virus] (537 aa)	Nuclprot1.1_WBvirCamb (1011 nt)	114 (41,68,2,-3)	75(1e-11)	2..337–149..262
			Nuclprot1.2_WBvirCamb (1139 nt)	311 (82,141,9,+ 1)	98(7e-19)	127..1032–127..420
			Nuclprot1.3_WBvirCamb (2942 nt)	353 (81,143,11,-3)	79(8e-12)	1267..2292–103..429
Xinzhou Mosquito Virus strain XC3–5	Viruses; ssRNA viruses; ssRNA negative-strand viruses; unclassified ssRNA negative-strand viruses.	gi|752455830|gb|AJG39271.1| RNA-dependent RNA polymerase [Xinzhou Mosquito Virus] (2022 aa)	RdRP_XinzhouVirCamb (8129 nt)	1401 (1076,1212,0,+ 1) 180 (118,144,0,+ 2) 392 (193,262,12,+ 3)	2189 (0.0) 241(4e-60) 389(e-105)	1300..5502–1..1401 5504..6043–1402..1579 6219..7358–1639..2019
Wuhan Mosquito Virus 1 strain WT3–15	Viruses; ssRNA viruses; ssRNA negative-strand viruses; unclassified ssRNA negative-strand viruses.	gi|752455822|gb|AJG39267.1| RNA-dependent RNA polymerase [Wuhan Mosquito Virus 1] (2095 aa)	RdRP1.1_Wuhan1virCamb (3576 nt)	1167 (716,918,1,+ 1)	1519 (0.0)	1..351–932..2070
			RdRP1.2_Wuhan1virCamb (2929 nt)	966 (625,749,0,+ 1)	1287 (0.0)	31..2928–1..966
			RdRP1.3_Wuhan1virCamb (3939 nt)	1300 (907,1072,0,+ 1)	1889 (0.0)	19..3918–49..1348
			RdRP1.5_Wuhan1virCamb (518 nt)	169 (78,113,0,+ 1)	166(3e-43)	10..516–1611..1779
			RdRP1.6_Wuhan1virCamb (6431 nt)	2095 (1287,1605,0,+ 3)	2670 (0.0)	63..6347–1..2094
			RdRP1.7_Wuhan1virCamb (6435 nt)	2096 (1327,1647,0,-2)	2737 (0.0)	81..6368–1..2095
		gi|752455880|gb|AJG39296.1|glycoprotein precursor [Wuhan Mosquito Virus 1] (681 aa)	GP1.1_Wuhan1virCamb (523 nt)	87 (48,75,0,+ 2)	116(1e-26)	92..352–589..675
			GP1.3_Wuhan1virCamb (1282 nt)	107 (70,85,0,+ 1)	160(4e-47)	781..1101–273..379
			GP1.5_Wuhan1virCamb (2231 nt)	652 (326,445,1,-3)	704 (0.0)	166..2118–24..675
			GP1.6_Wuhan1virCamb (2205 nt)	675 (326,473,1,+ 1)	717 (0.0)	22..2043–1..675
			GP1.7_Wuhan1virCamb (2219 nt)	640 (344,466,1,+ 1)	740 (0.0)	127..2043–36..675
		gi|752455945|gb|AJG39330.1| nucleopasid protein [Wuhan Mosquito Virus 1] (345 aa)	NuclCap1.1_Wuhan1virCamb (645 nt)	210 (122,150,0,+ 1)	234(6e-72)	16..645–43..249
			NuclCap1.2_Wuhan1virCamb (735 nt)	174 (107,131,0,+ 1)	211(2e-62)	214..735–23..193
			NuclCap1.3_Wuhan1virCamb (629 nt)	205 (122,149,0,+ 2)	231(7e-71)	14..628–38..239
			NuclCap1.4_Wuhan1virCamb (546 nt)	103 (53,68,0,+ 3)	110(2e-25)	3..311–228..330
			NuclCap1.5_Wuhan1virCamb (549 nt)	168 (86,109,0,+ 3)	156(2e-42)	45..548–2..165
			NuclCap1.7_Wuhan1virCamb (1259 nt)	160 (81,110,0,+ 1)	168(5e-44)	1..480–174..333
			NuclCap1.8_Wuhan1virCamb (1015 nt)	240 (126,162,7,+ 1)	228(6e-68)	316..1014–6..241
			NuclCap1.9_Wuhan1virCamb (3081 nt)	310 (172,218,0,+ 1)	332(e-100)	1213..2142–2..307
			NuclCap1.10_Wuhan1virCamb (1473 nt)	342 (178,229,0,+ 3)	339(e-108)	366..1391–6..336
			NuclCap1.11_Wuhan1virCamb (1791 nt)	332 (178,225,0,-3)	343(e-109)	782..1777–6..333
			NuclCap1.12_Wuhan1virCamb (2147 nt)	346 (184,230,1,+ 3)	345(e-108)	357..1391–6..343
Wuhan Mosquito Virus 9 strain JX1–13	Viruses; ssRNA viruses; ssRNA negative-strand viruses; unclassified ssRNA negative-strand viruses.	gi|752455734|gb|AJG39217.1| glycoprotein [Wuhan Mosquito Virus 9] (614 aa)	GP1.1_Wuhan9virCamb (658 nt)	164 (43,86,1,-1)	71(6e-11)	2..490–50..210
			GP1.2_Wuhan9virCamb (924 nt)	130 (31,63,2,+ 1)	58(5e-06)	232..615–101..230
			GP1.3_Wuhan9virCamb (2429 nt)	89 (20,43,0,+ 2) 353 (82,148,6,+ 1)	41(1e-23) 99(1e-23)	590..856–51..139 841..1881–135..441
		gi|752455731|gb|AJG39214.1| ORF1 [Wuhan Mosquito Virus 9] (279 aa)	ORF1.1_Wuhan9virCamb (1872 nt)	192 (55,91,2,+ 1)	85(9e-15)	1012..1581–13..198
			ORF1.2_Wuhan9virCamb (1625 nt)	90 (27,49,2,+ 1)	55(4e-05)	1075..1338–13..102
			ORF1.3_Wuhan9virCamb (1202 nt)	205 (56,96,2,+ 2)	85(3e-15)	515..1123–13..211
Xincheng Mosquito Virus strain XC1–6	Viruses; ssRNA viruses; ssRNA negative-strand viruses; unclassified ssRNA negative-strand viruses	gi|752455743|gb|AJG39224.1| ORF1 [Xincheng Mosquito Virus] (462 aa)	ORF1_XinchengVirCamb (1329 nt)	28 (11,15,0,+ 2) 155 (62,92,16,+ 3) 97 (37,57,1,+ 1)	27(1e-36) 105(1e-36) 70(1e-36)	227..310–155..182 339..755–194..346 769..1056–351..445
		gi|752455745|gb|AJG39226.1| glycoprotein [Xincheng Mosquito Virus] (648 aa)	GP1.1_XinchengVirCamb (509 nt)	132 (56,80,0,+ 2)	120(3e-28)	5..400–421..552
			GP1.2_XinchengVirCamb (953 nt)	242 (98,147,1,+ 2)	203(5e-56)	50..772–139..379
			GP1.3_XinchengVirCamb (1635 nt)	119 (39,67,9,+ 2) 143 (49,74,17,+ 3)	79(6e-12) 85(4e-14)	491..820–244..362 639..1016–303..445
			GP1.5_XinchengVirCamb (1313 nt)	100 (63,80,0,+ 2) 165 (106,135,0,+ 3)	150(3e-36) 232(3e-65)	1010..1309–190..289 546..1040–290..450
			GP1.6_XinchengVirCamb (3076 nt)	402 (176,254,14,+ 2)	362(e-108)	1814..2977–230..631
			GP1.7_XinchengVirCamb (1314 nt)	350 (164,242,0,+ 3)	371(e-118)	228..1277–268..617
			GP1.8_XinchengVirCamb (2660 nt)	627 (242,370,36,+ 2)	475(e-152)	482..2254–6..624
			GP1.9_XinchengVirCamb (1757 nt)	65 (32,49,0,+ 2) 394 (246,306,6,+ 1)	83(2e-13) 523(e-174)	29..223–173..237 226..1389–236..629
		gi|752455746|gb|AJG39227.1| RNA-dependent RNA polymerase [Xincheng Mosquito Virus] (2026 aa)	RdRP1.1_XinchengVirCamb (925 nt)	306 (137,185,5,+ 2)	240(4e-67)	23..925–270..574
			RdRP1.2_XinchengVirCamb (904 nt)	28 (20,23,0,+ 1) 61 (54,56,0,+ 3) 202 (141,161,5,+ 2)	45(6e-28) 107(6e-28) 288(9e-84)	127..210–474..501 210..392–503..563 281..871–536..728
			RdRP1.3_XinchengVirCamb (991 nt)	241 (105,141,4,+ 3)	190(2e-49)	213..923–159..399
			RdRP1.4_XinchengVirCamb (1065 nt)	115 (85,100,0,+ 1) 64 (50,56,0,+ 2) 73 (52,64,0,+ 3) 48 (34,43,0,+ 1) 52 (42,48,0,+ 2)	179(e-133) 103(e-133) 120(e-133) 75(e-133) 88(e-133)	13..357–835..949 365..556–952..1015 549..767–1012..1084 766..909–1085..1132 908..1063–1166..1217
			RdRP1.5_XinchengVirCamb (1354 nt)	431 (204,278,1,+ 1)	401(e-121)	64..1353–587..1013
			RdRP1.6_XinchengVirCamb (2062 nt)	316 (216,249,0,+ 1)	447(e-134)	1114..2061–359..671
			RdRP1.7_XinchengVirCamb (3974 nt)	29 (26,27,0,+ 3) 608 (422,501,0,+ 1) 479 (274,351,0,+ 2) 96 (58,76,0,+ 1) 63 (34,43,0,+ 3)	62(5e-06) 883 (0.0) 568 (0.0) 130 (0.0) 76 (0.0)	24..110–730..758 100..1923–755..1361 1898..3334–1352..1830 3343..3630–1836..1931 3642..3830–1955..2017
Nienokoue virus isolate B51/CI/2004	Viruses; ssRNA viruses; ssRNA positive-strand viruses, no DNA stage; Flaviviridae	gi|655454925|ref.|YP_009041466.1| polyprotein [Nienokoue virus] (3356 aa)	PolProt1.1_FlavivirusCamb (1008 nt)	265 (81,115,2,+ 2)	111(6e-23)	203..991–1322..1578
			PolProt1.2_FlavivirusCamb (2193 nt)	439 (184,264,4,+ 2) 96 (40,58,0,+ 2) 30 (17,23,0,+ 1)	329(3e-92) 79(6e-19) 45(6e-19)	32..1336–1675..2108 1385..1672–1529..1623 1738..1827–1638..1667
			PolProt1.3_FlavivirusCamb (1010 nt)	137 (67,86,0,+ 1)	135(1e-30)	565..975–1855..1990
			PolProt1.4_FlavivirusCamb (10610 nt)	3342 (1354,1889,63,-3)	2383 (0.0)	514..10350–92..3348
Tobacco streak virus isolate pumpkin segment RNA1	Viruses; ssRNA viruses; ssRNA positive-strand viruses, no DNA stage; Bromoviridae; Ilarvirus	gi|254554401|gb|ACT67442.1| replicase [Tobacco streak virus] (1092 aa)	Replicase_TSvirCamb (1565 nt)	308 (83,136,8,+ 1)	95(3e-17)	364..1263–39..309
Oat golden stripe virus RNA1	Viruses; ssRNA viruses; ssRNA positive-strand viruses, no DNA stage; Virgaviridae; Furovirus	gi|9635455|ref.|NP_059511.1| replicase [Oat golden stripe virus]	Replicase_OatGSvirCamb (1661 nt)	320 (85,143,28,+ 3)	107(1e-20)	666..1541–18..327

**Table 3:** Similarity of Senegal and Cambodia virus assemblies by BLASTX to 24 reference viruses in GenBank. Ten targets are shared, nine are Senegal-specific, and five are Cambodia-specific

Reference virus	Viral taxonomy	Senegal libraries	Cambodia libraries
Culex tritaeniorhynchus rhabdovirus RNA, complete genome	Viruses; ssRNA viruses; ssRNA negative-strand viruses; Mononegavirales; Rhabdoviridae; unclassified Rhabdoviridae.		
Phasi Charoen-like virus	Viruses; ssRNA viruses; ssRNA negative-strand viruses; Bunyavirales; unclassified Bunyavirales.		
Uncultured virus isolate acc_1.3	Viruses; environmental samples.		
Wellfleet Bay virus isolate 10–280-G segment 4	Viruses; ssRNA viruses; ssRNA negative-strand viruses; Orthomyxoviridae; Quaranjavirus; unclassified Quaranjavirus.		
Wuhan Mosquito Virus 1 strain WT3–15	Viruses; ssRNA viruses; ssRNA negative-strand viruses; unclassified ssRNA negative-strand viruses.		
Wuhan Mosquito Virus 9 strain JX1–13	Viruses; ssRNA viruses; ssRNA negative-strand viruses; unclassified ssRNA negative-strand viruses.		
Xincheng Mosquito Virus strain XC1–6	Viruses; ssRNA viruses; ssRNA negative-strand viruses; unclassified ssRNA negative-strand viruses.		
Xinzhou Mosquito Virus strain XC3–5	Viruses; ssRNA viruses; ssRNA negative-strand viruses; unclassified ssRNA negative-strand viruses.		
Beaumont virus strain 6	Viruses; ssRNA viruses; ssRNA negative-strand viruses; Mononegavirales; Rhabdoviridae; unclassified Rhabdoviridae.		
Jurona virus	Viruses; ssRNA viruses; ssRNA negative-strand viruses; Mononegavirales; Rhabdoviridae; Vesiculovirus.		
Omono River virus	Viruses; dsRNA viruses		
American dog tick phlebovirus isolate FI3	Viruses; ssRNA viruses; ssRNA negative-strand viruses; Bunyavirales; Phlebovirus; unclassified Phlebovirus.		
Daeseongdong virus 1 strain A12.2708/ROK/2012	Viruses; unclassified viruses.		
DsRNA virus environmental sample clone mill.culi_contig84	Viruses; dsRNA viruses; environmental samples.		
Homalodisca vitripennis reovirus segment S3	Viruses; dsRNA viruses; Reoviridae; Sedoreovirinae; Phytoreovirus; unclassified Phytoreovirus		
Ixodes scapularis associated virus 2 isolate A1, partial genome	Viruses; unclassified viruses.		
Sunn-hemp mosaic virus	Viruses; ssRNA viruses; ssRNA positive-strand viruses, no DNA stage; Virgaviridae; Tobamovirus.		
Uncultured virus isolate acc_7.4	Viruses; environmental samples.		
Wuhan Spider Virus strain SYZZ-2	Viruses; ssRNA viruses; ssRNA negative-strand viruses; unclassified ssRNA negative-strand viruses.		
Nienokoue virus isolate B51/CI/2004	Viruses; ssRNA viruses; ssRNA positive-strand viruses, no DNA stage; Flaviviridae		
Oat golden stripe virus RNA1	Viruses; ssRNA viruses; ssRNA positive-strand viruses, no DNA stage; Virgaviridae; Furovirus		
Puerto Almendras virus isolate LO-39	Viruses; ssRNA viruses; ssRNA negative-strand viruses; Mononegavirales; Rhabdoviridae; unclassified Rhabdoviridae.		
Tobacco streak virus isolate pumpkin segment RNA1	Viruses; ssRNA viruses; ssRNA positive-strand viruses, no DNA stage; Bromoviridae; Ilarvirus		
Bivens Arm virus isolate UF 10	Viruses; ssRNA viruses; ssRNA negative-strand viruses; Mononegavirales; Rhabdoviridae; Tibrovirus		

**Fig. 2:**
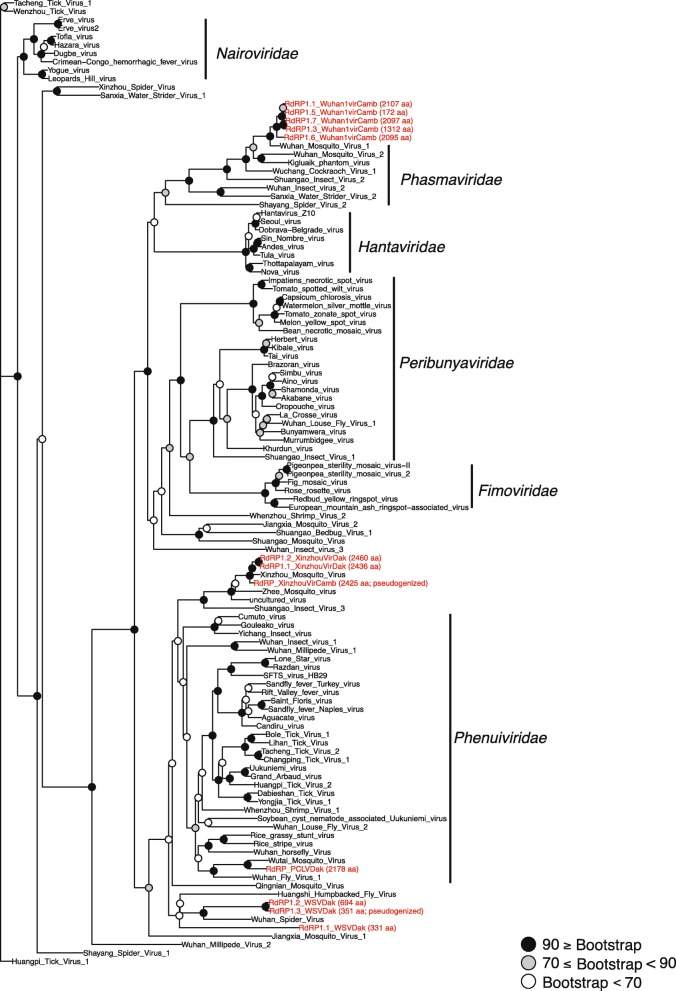
Phylogenetic tree of reference and novel virus assemblies from the *Bunyavirales* order. Maximum-likelihood phylogeny based on RNA-dependent RNA polymerase (RdRP) predicted peptide sequences of viruses from the *Bunyavirales* order. Novel viruses characterized in the current study (red name labels) are placed with reference viruses (black name labels) within the Phasmavirus clade and in a basal position of the Phlebovirus-Tenuivirus clade. Node robustness is indicated by bootstrap values (number of replicates supporting the node), indicated by color of the dot at the branch base, see key. Protein lengths and functional status of RdRP peptide sequences from novel viruses in the current study are included to distinguish between complete and partial and/or non-functional pseudogenes (indicated by label “pseudogenized”, functional status also shown in Additional file 2: Table S2 and Additional file 3: Table S3). Average protein size of reference virus RdRP genes is 2496 amino acids

**Fig. 3:**
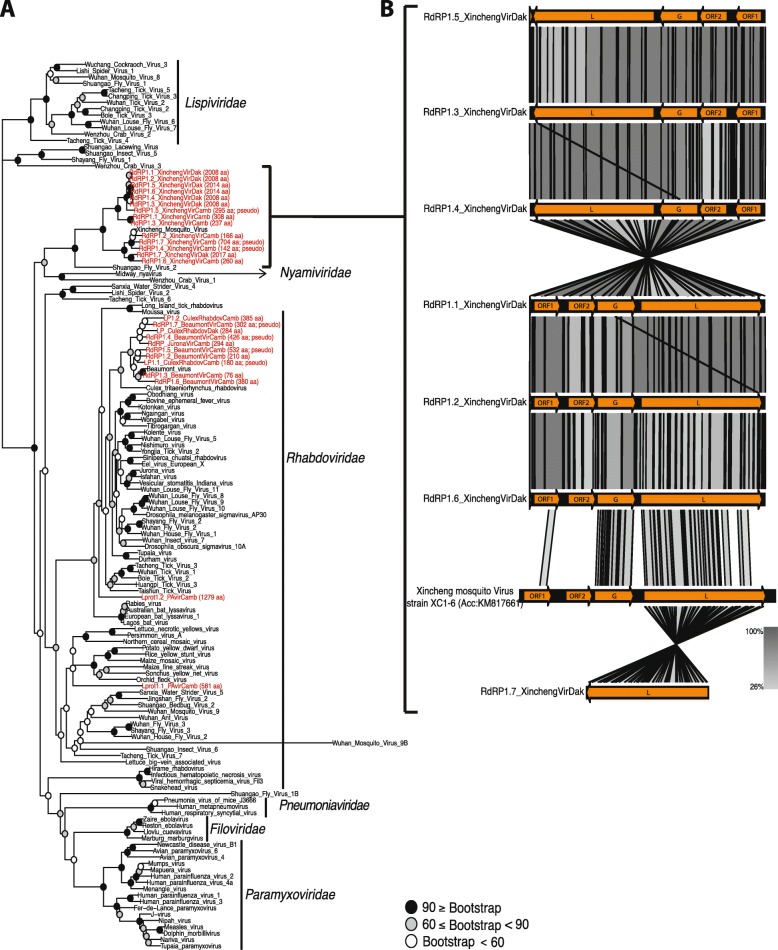
Phylogenetic tree of reference and novel virus assemblies from the *Mononegavirales* order. **a** Maximum-likelihood phylogeny based on RNA-dependent RNA polymerase (RdRP) predicted peptide sequences of viruses from *Mononegavirales* order. Novel virus assemblies characterized from Cambodia and Senegal *Anopheles* samples (red name labels) are placed with reference viruses (black name labels), predominantly within the Dimarhabdovirus clade and as close relative of the Nyamivirus clade. Node robustness is indicated by bootstrap values (number of replicates supporting the node), indicated by color of the dot at the branch base, see key. Protein lengths and functional status of RdRP peptide sequences from novel viruses in the current study are included to distinguish between complete and partial and/or non-functional pseudogenes (indicated by label “pseudo”, functional status indicated in Additional file 2: Table S2 and Additional file 3: Table S3). Average protein size of reference virus RdRP genes is 2098 amino acids. **b** Genome comparison of novel and reference Xincheng Mosquito Viruses, which are too diverged to align at the nucleic acid sequence level. Grey blocks represent peptide sequence homology regions between compared sequences. The nucleotide sequences of the entire viral contigs, and not only of the RdRP gene as in (**a**), were translated and used to search the translated nucleotide database with TBLASTX. The viruses display recognizable relatedness over their genomes, despite geographic distance and nucleotide sequence divergence. Color intensity indicates identity levels from TBLASTX results (values indicated in key)

In order to place these 115 novel virus assemblies in an evolutionary context, phylogenetic trees were constructed from predicted peptide sequences of conserved regions of the RNA-dependent RNA polymerase (RdRP) gene annotated in the 115 virus sequences, along with RdRP peptide sequences of related virus sequences from GenBank. This allowed the placement of 44 of the 115 assembled viruses in phylogenetic trees, revealing clusters of highly related viruses in the wild *Anopheles*. Notable examples include five novel virus assemblies from Cambodian *Anopheles* placed near Wuhan Mosquito Virus 1 in a monophyletic group of the *Phasmaviridae* family (*Bunyavirales*) (Fig. 2). In addition, within the order *Mononegavirales*, 14 novel *Anopheles* virus assemblies (7 from Cambodia and 7 from Senegal) formed a monophyletic group that includes Xincheng Mosquito Virus and Shungao Fly Virus. Finally, 10 novel virus assemblies (9 from Cambodia, 1 from Senegal) formed a monophyletic group that includes Beaumont Virus and a virus from *Culex tritaeniorhynchus* within the *Rhabdoviridae* family (Fig. 3a).

We characterized the degree of completeness of the virus assemblies to determine whether they contain full or almost full viral genome sequences, and whether predicted peptides are likely to be functional. Many of the viral contigs are too diverged from each other and from reference viruses in the phylogenetic tree to align informatively at the nucleotide level, and reliable sequence comparisons were only possible at the peptide sequence level. We translated nucleotide sequences for the novel viral contigs and compared them to the translated nucleotide database using TBLASTX. An example of this analysis is shown for viral contigs homologous to Xincheng virus (Fig. 3b). Closely-related viral contigs (for example RdRP1.7_XinchengVirDak and RdRP1.3_XinchengVirDak), with on average 95% nucleotide identity over the full contig length, can thus be compared with the more divergent viral contigs such as RdRP1.7_XinchengVirDak, which does not align at the nucleotide level with the first two, but does align when translated to peptide sequences.

This combined nucleotide and peptide-based analysis was applied to the 115 novel viral contigs. A total of 195 open reading frames (ORFs) were annotated among the 115 viral contigs, an average of 1.7 ORFs per viral contig (Additional file 2: Table S2 and Additional file 3: Table S3). Based on TBLASTX alignments with the closest reference viral genomes, 56 of the 195 ORFs, found in 25 of the viral contigs, appear to be fragmented or frameshifted ORF sequences potentially associated with pseudogenes, as compared to the complete gene present in the homologous reference virus, indicating a possible non-functional status for these 25 contigs. Four additional viral contigs contained small ORFs as compared to the cognate gene in the closest annotated reference viral genomes. In contrast, 67 of the 195 ORFs were complete, and 68 ORFs were partial for technical reasons, because of fragmented viral assemblies that do not cover the entire viral gene (Additional file 3: Table S3).

Thus, peptide comparisons with reference viral genomes provided evidence supporting a functional status for 86 of the 115 novel viral contigs, while 29 of the contigs displayed a potential non-functional status. The source of these latter 29 viral contigs is unknown, but they display equivalent sequence representation and assembly quality as the 86 contigs. They most likely represent functional viruses that engage in programmed ribosomal frameshifting or transcriptional slippage [15, 16], which has been reported for at least flavivirus and alphavirus ISVs [17, 18]. For these otherwise high-quality viral contigs with frameshifts or short ORFs, further work would be necessary to distinguish between hypotheses of transcriptional slippage, ORFs under relaxed selection pressure, or technical error. However, overall we find high levels of collinearity and similarity among novel viruses at the protein level that are not necessarily matched by comparable levels of similarity at the nucleotide level. These comparisons revealed potential populations of closely related but diverged viruses colonizing *Anopheles* from widely separated geographic locations, in some cases with different degrees of divergence over the same genomic region.

### Quantification of novel virus sequences in mosquito samples

**Fig. 4:**
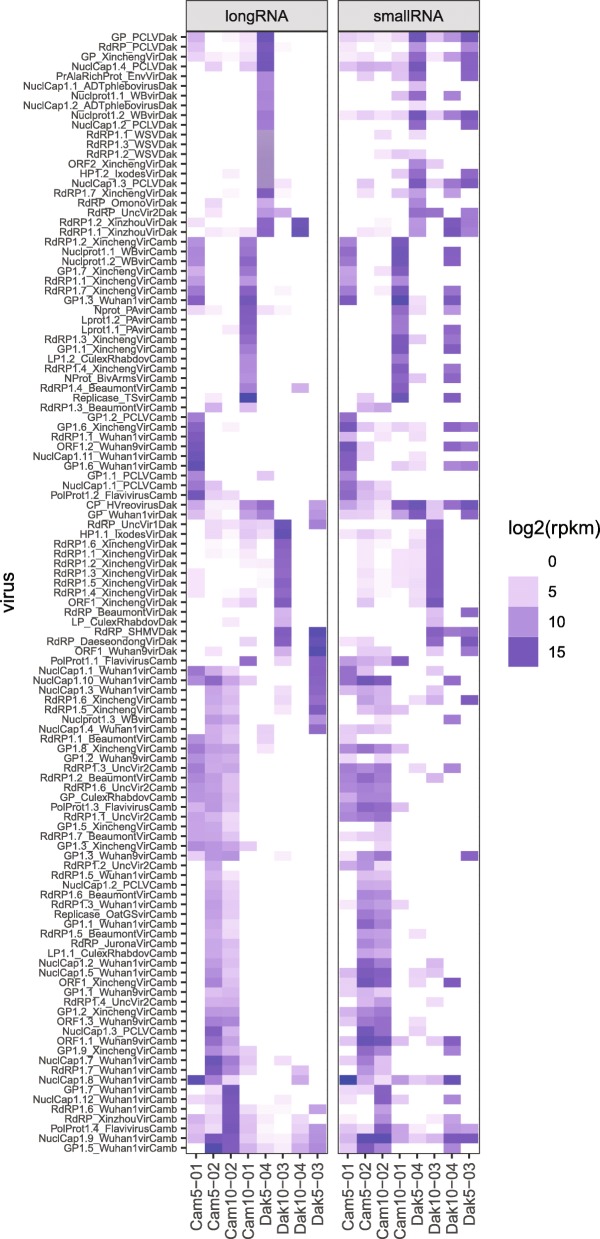
Virus abundance profiles across mosquito sample pools based on long and small RNA sequence mapping. Heatmap of log2-transformed reads per kilobase per million reads (RPKM) abundance values of novel virus assemblies identified from Cambodia and Senegal sample pools based on long and small RNA sequence libraries. Broadly similar viral abundance profiles are detected in sample pools by the long and small RNA sequence data. Representation of particular viruses is uneven among mosquito sample pools, suggesting inter-individual mosquito differences for virus carriage. X-axis, *Anopheles* sample pools from Cambodia, Cam, and Senegal, Dak; y-axis, names of 115 assembled virus contigs displaying sequence similarity to known virus families (Additional file 2: Table S2 and Additional file 3: Table S3)

In order to evaluate the prevalence of novel virus sequences across the analyzed mosquito samples, host-filtered small and long RNA reads were mapped over the 115 novel virus sequences identified by de novo sequence assembly. Based on long RNAseq reads, the abundance profiles of the 115 virus assemblies display a non-overlapping distribution across different sample pools of 5 or 10 mosquitoes per pool, and virus sequences can be localized to particular sample pools from the abundance profiles (Fig. 4, left panel). This probably indicates a patchy prevalence and abundance of the different viruses among individual mosquitoes, such that an individual mosquito highly infected with a given virus could potentially generate a strong signal for that virus in the sample pool. The sample pools from Cambodia share a higher fraction of common viruses, while there is less overlap in virus abundance distribution across sample pools from Senegal. The representation of virus distribution based on small RNA sequence reads displayed profiles broadly similar to the long RNA-based abundance distribution (Fig. 4, right panel). This observation is consistent with the expectation that small RNA representation is a signature of virus double-stranded RNA (dsRNA) processing by the mosquito RNA interference (RNAi) machinery [19], and was examined next.

### Small RNA size profiling

**Fig. 5:**
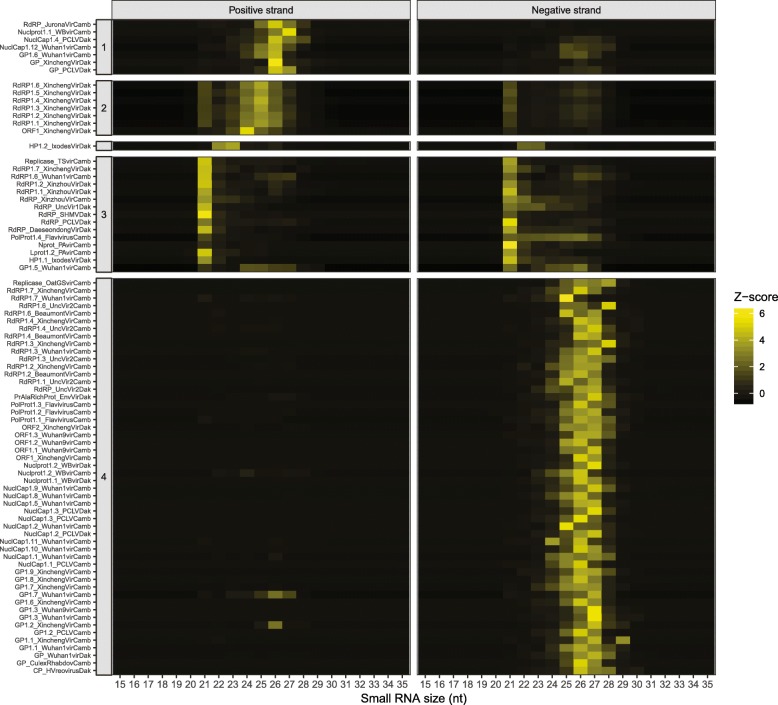
Small RNA size profiles of novel virus assemblies from Cambodia and Senegal sample pools. Hierarchical clustering of 88 novel virus assemblies based on Pearson correlation of small RNA size profiles. The 88 viruses were the members of the 115 novel virus set meeting the threshold of at least 100 small RNA reads mapped to the viral contig, to assure reliable small RNA size profiling. Small RNA reads that mapped to each of the 88 virus assemblies were extracted, and their size distributions were normalized with a z-score transformation. Heatmaps indicate the frequency of small RNA reads of size 15 to 35 nucleotides that map over the positive strand (left panel) and negative strand (right panel) of the reference sequence indicated on the y-axis. The x-axis indicates the size in nucleotides of the small RNAs mapped. Four main clusters were defined (indicated by numbers on the left of each panel) based on these small RNA size profiles. The profile in Cluster 3 is enriched for 21 nucleotide reads mapping over both positive and negative strands, characteristic of the classical small interacting RNA (siRNA) product size profile

The processing of virus sequences by small RNA pathways of the insect host generates diagnostic patterns of small RNA read sizes from different viruses. In order to evaluate this phenomenon we first imposed a threshold of at least 100 small RNA reads mapped to the viral contig, to assure reliable small RNA size profiling, and 82 of the 115 novel virus assemblies were retained for the analysis. Small RNA reads that mapped to each of the 82 virus assemblies were extracted, and their size distributions were normalized with a z-score transformation. This allowed comparison of the z-score profiles among virus assemblies by pairwise correlation analysis and hierarchical clustering. The relationship between the small RNA profiles of the different viruses could then be visualized as a heatmap. The results of this analysis revealed the presence of four major groups of virus sequences based on small RNA size profiles (Fig. 5). Cluster 1 consists of 7 virus assemblies generating small RNAs predominantly in the size range of 23–29 nt mapping over the positive, and to a lesser extent negative, strand. Cluster 2 includes 7 viruses, all from Senegal, and displays a similar size profile as viruses of Cluster 1 with reads in the 23–29 nt size range, but also with a higher frequency of 21 nt reads mapping over the positive and negative strands, emblematic of virus cleavage through the mosquito host RNAi pathway. Cluster 3 includes 15 viruses that exhibit the classic pattern of viral RNA processing by the host RNAi pathway, with reads predominantly of 21 nt in length mapping over virus positive and negative strands (small RNA size and coverage profiles for this Cluster shown in Additional file 4: Figure S1). Finally, Cluster 4 includes 52 viruses with small RNA size profiles dominated by reads of 23–29 nt mapping predominantly over the negative strand of virus sequences. Because of the strong strand bias of small RNAs observed, this pattern could correspond to degradation products of virus RNAs, although alternatively, there appears to be size enrichment in the 27–28 nt size peaks characteristic of PIWI-interacting RNAs (piRNAs), and we examine this possibility below using bioinformatic and functional analyses.

### Viral origin of unclassified transcripts by small RNA size profiling

A major drawback of sequence similarity-based identification of novel viruses in de novo sequence assemblies is the dependence of detection upon existing records of close relatives in public databases. Aguiar et al. proposed that the small RNA size profiles of arthropod-derived viruses detected by sequence similarity could be used as signature to recruit unclassified contigs from de novo sequence assemblies of potential viral origin [19]. We implemented this strategy in order to identify additional sequences of putative viral origin in the set of 2114 contigs left unclassified by sequence similarity searching but meeting the same quality criteria as the 115 contigs (non-redundant and > 500 nucleotides), and with at least 100 small RNA sequence reads.

Of these unclassified contigs, a likely viral origin is supported for 4 and 35 contigs that display strong association by small RNA profile with Cluster 2 and Cluster 3, respectively (Spearman correlation> 0.9, Additional file 5: Figure S2). These clusters display small RNA size profiles mapping to both genome strands, which are characteristic of classic RNAi processing of viral dsRNA replication intermediates. Thus, in addition to the 115 novel virus assemblies classified by sequence similarity to known viruses, 39 unclassified high-quality novel *Anopheles* virus assemblies were identified, without sequence similarity to identified viruses. Further work will be necessary to characterize the biology of these unclassified novel virus assemblies.

Of the other assemblies unclassified by sequence similarity, 1566 showed strong associations between their small RNA size profiles and the small RNA size profiles of virus contigs detected by sequence similarity (Spearman correlation> 0.9). Among these, the majority were associated with Cluster 4 virus assemblies (1219 unclassified contigs) and to less extent with Cluster 1 (309 unclassified contigs). Both clusters were characterized by a strong bias towards reads from a single strand (positive for Cluster 1 and negative for Cluster 4).

To evaluate how specific these latter profiles of 1219 and 309 contigs are for virus-related sequences, we designed a reconstruction control experiment using the same small RNA size profiling and clustering analysis as above, but instead using 669 RNA contigs known to map to the mosquito reference assembly, thus strictly of host origin. As above, contigs with at least 100 small RNA sequence reads were used. Five hundred sixty-one of these mosquito contigs could be grouped with small RNA size profiles of virus contigs (Spearman correlation> 0.9), most of them (98.21%) with Cluster 4 (78.6%) and Cluster 1 (19.6%) profiles.

### *Anopheles* may produce piRNAs from the RNA virome

piRNAs are endogenous small noncoding RNAs that ensure genome stability by protecting it from invasive transposable elements such as retrotransposons and repetitive or selfish sequences [20]. In addition, in *Aedes* mosquito cells, piRNAs can probably mediate responses to arboviruses or ISVs [20–23]. *Anopheles* mosquitoes express annotated piRNAs from genomic piRNA clusters [24, 25]. The small RNAs in Clusters 1 and 4 display a strand bias, and many somatic piRNAs also map to only one strand in *Drosophila* and other arthropods [20, 26]. Notably, many virus-related piRNAs in *Aedes*, which are largely ISV-derived, mainly map only to the virus strand antisense to the viral ORF [22].

In *An. coluzzii*, about half of annotated piRNAs display a strong or exclusive strand bias [25], which is a greater proportion of unidirectional piRNAs than *Drosophila*. Until the current study, *Anopheles* piRNAs have not previously been examined for relatedness to ISVs. Overall, these small RNA results are probably most consistent with an interpretation that RNA profile Cluster 1 and Cluster 4 detect strand-biased piRNAs derived from the natural ISV virome of wild *Anopheles*. On that interpretation, the above 561 contigs mapping to host that share the Cluster 1 and Cluster 4 RNA profiles are most likely also piRNAs, but instead derived from endogenous host RNA templates. Previous results showed that most *An. coluzzii* piRNAs are derived from long-terminal repeat retrotransposons and DNA transposable elements [25]. Our current results add wild ISVs as a possible source of template for *Anopheles* piRNA production, and indicate that further work is warranted on *Anopheles* piRNA. Our results also suggest the possibility that piRNAs may be involved in *Anopheles* response to viruses, a phenomenon found for only *Aedes* among a wide range of arthropods [20], but *Anopheles* were not tested.

### O’nyong nyong alphavirus infection influences expression of piRNAs in *Anopheles coluzzii*

The potential that *Anopheles* piRNAs could be involved in response or protection to virus infection has not been previously examined or reported to our knowledge. To examine this possibility, we challenged *An. coluzzii* mosquitoes with the alphavirus ONNV by feeding an infectious bloodmeal, and sequenced small RNAs expressed during the primary infection at 3 d post-bloodmeal. Mosquitoes fed a normal bloodmeal were used as the control condition.

The small RNAs were mapped to previously annotated *An. coluzzii* candidate piRNA genes located in 187 genomic piRNA clusters [25], and expression levels of the piRNA genes in response to ONNV infection were tested using Cuffdiff. The analysis detected 86 piRNA genes displaying differential abundance levels between ONNV infected mosquitoes and normal bloodmeal controls (Additional file 6: Table S4). Filtering these 86 genes on quality criteria of i) length of the contiguous region expressed in small RNA < 40 nt, characteristic of piRNA size, and ii) normalized read depth in the upper 10% for the most robust signals, highlighted just two annotated piRNA candidates, XLOC_012931 and XLOC_012762. Both candidate piRNAs displayed significantly lower abundance in small RNA after ONNV infection as compared to uninfected controls, suggesting that these two piRNAs were downregulated during ONNV infection (locus XLOC_012931, Cuffdiff test statistic = 3.23, *p*-value = 5e-5, adjusted *p*-value = 6.7e-3, reference genome sequence coordinates AgamP4:UNKN:19043685:19043716; and locus XLOC_012762, Cuffdiff test statistic = 2.39, *p*-value = 9.5e-4, adjusted *p*-value = 0.046, reference genome sequence coordinates AgamP4:UNKN:13088289:13088321).

**Fig. 6:**
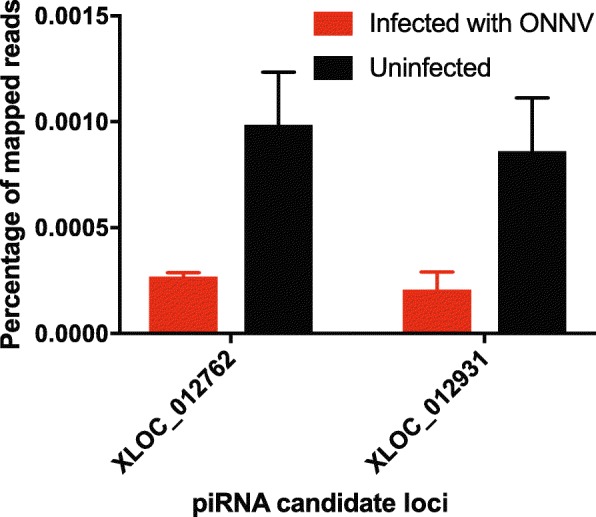
O’nyong nyong arbovirus infection influences expression of candidate piRNA genes in *Anopheles coluzzii*. *Anopheles coluzzii* mosquitoes were challenged with O’nyong nyong virus (ONNV) by feeding an infectious bloodmeal or an uninfected control bloodmeal, and small RNAs expressed during the primary infection at 3 d post-bloodmeal were sequenced. Analysis using Cuffdiff highlighted two candidate piRNA genes that displayed decreased abundance of mapped small RNAs in ONNV infected samples (see Results, piRNA loci XLOC_012931 and XLOC_012762). Here, the small RNA sequence reads mapping to the two candidate piRNA loci were quantified using the Integrative Genomics Viewer normalized to the library size, and the difference between ONNV infected and uninfected samples tested statistically. X-axis indicates candidate piRNA locus, y-axis indicates percentage of normalized small RNA reads mapping to the piRNA gene. ONNV-infected mosquitoes, red bar; uninfected control mosquitoes, black bar. Experiments were done in two biological replicates, error bars indicate standard deviation. Locus XLOC_012931, Chi-squared = 77.36, df = 1, *p*-value< 2.2e-16 (ONNV-infected mean mapped reads = 36 ± 141,421,356, mean total reads = 19,193,551 ± 8,555,908.61, ONNV-uninfected mean mapped reads = 160 ± 14,1,421,356, mean total reads = 19,167,336 ± 3,962,902.88052); and locus XLOC_012762, Chi-squared = 75.78, df = 1, *p*-value< 2.2e-16 (ONNV-infected mean mapped reads = 51 ± 19,09, mean total reads = 19,193,551 ± 8,555,908.61, ONNV-uninfected, mean mapped reads = 184 ± 848,528,137, mean total reads = 19,167,336 ± 3,962,902.88)

Differential abundance was confirmed by quantifying small RNAs mapping to the two candidate piRNAs using the Integrative Genomics Viewer. By this test also, both candidate piRNAs displayed lower normalized read counts in ONNV infected samples as compared to uninfected controls (Fig. 6; locus XLOC_012931, Chi-squared = 77.36, df = 1, *p*-value< 2.2e-16; and locus XLOC_012762, Chi-squared = 75.78, df = 1, *p*-value< 2.2e-16).

## Discussion

The current study contributes to a growing body of work defining the deep diversity of the invertebrate virosphere [14, 27, 28]. Because mosquitoes transmit viral infections of humans and animals, there is particular interest in discovery of ISVs comprising the mosquito virome [6, 29–31]. Here, we sampled *Anopheles* mosquitoes from two zones of forest exploitation that are considered disease emergence zones with likely exposure of the human and domestic animal populations to sylvan pathogens. Using assembly quality criteria of non-redundant contigs at least 500 nt in length, we identified 115 novel RNA virus assemblies by sequence similarity to known virus families, and an additional 39 high-confidence virus assemblies that were unclassified by sequence similarity, but display characteristic products of RNAi processing of replication intermediates. Finally, 1566 unclassified contigs possessed comparable assembly quality, and lacked a strong RNAi processing signature, but displayed a signature consistent with piRNA origin. This latter group will require additional work to filter genuine virus-derived piRNA sequences, which have been previously reported in *Aedes* mosquitoes [20–23], from other potential sources of piRNAs such as retrotransposons and DNA transposable elements, as well as possible physical degradation.

Taken together, at least 115 novel and non-redundant virus assemblies, and possibly many more, were identified in wild *Anopheles* mosquitoes in the current report. Small and long RNAs were sequenced from pools of 5–10 mosquitoes. Pooled sample analysis obscures the distribution and abundance of viruses among individuals in the population. Individual mosquito analysis will become a research priority as sequencing costs drop, and is the best way to determine ISV distribution and prevalence. However, some insight on virus distribution can be gained from the comparison of sample pools collected from the same site, for example Senegal or Cambodia. The abundance heatmap shown in Fig. 4 indicates that virus diversity is high in the *Anopheles* population, while the distribution of particular viruses is relatively uneven when comparing across *Anopheles* sample pools. This suggests that the number of viruses per individual is probably also low, leading to a patchy distribution of particular viruses among individuals. This is consistent with observations in our laboratory from individual mosquito sequencing and de novo assembly, which typically identifies < 5 distinct viruses per individual. We cannot exclude the presence of contaminating environmental viruses in the sequence set, for example adhered to the adult mosquito cuticle. Nevertheless, the samples were all washed, and if present, environmental virus contaminants would likely be rare, and would have been discarded early in the assembly pipeline because they would not contribute enough sequence reads to generate long assemblies to meet the quality threshold.

The dynamics of the virome may thus be different from the bacterial microbiome, in which at least tens of taxa are typically present per individual, and microbial diversity is thought to lead to homeostasis or resilience of the microbiota as an ecosystem within the host [32, 33]. By comparison, very little is known about the function of the mosquito virome within the host. At least three important topics are worth exploring.

First, unlike the bacterial microbiota, the stability and resilience over time of the viral assemblage in an individual mosquito is unknown. Members of the virome could persist in individual host populations over time in commensal form, or the uneven and patchy viral distribution observed among sample pools could be a consequence of successive waves of epidemic infection peaks passing through local populations. The commensal or epidemic models would have distinct biological implications for the potential influence of the virome, including on host immunity and competence for transmission of pathogens.

Second, the individual and population-level effect of ISV carriage on vector competence for pathogen transmission is a key question. In the current study, the predominant host species sampled are *Anopheles* vectors of human malaria, and in Africa, these species are also known or likely vectors of ONNV. ISVs have not been tested for influence on *Plasmodium* or ONNV infection in *Anopheles*, to our knowledge. ISVs could affect host immunity and malaria susceptibility, or even cause temporary vector population crashes during a putative ISV epidemic. A similar concept may apply to ISV interactions with the mosquito host for arbovirus transmission [30]. We identified relatives of Phasi Charoen-like virus (PCLV) in *Anopheles* from Senegal and Cambodia. PCLV relatives also infect *Aedes*, where they reduced the replication of ZIKV and DENV arboviruses [34]. Palm Creek virus, an insect specific flavivirus, caused reduced replication of the West Nile virus and Murray Valley encephalitis arboviruses in *Aedes* cells [35]. Clearly, ISV co-infection of mosquito vectors with *Plasmodium* and/or arboviruses in nature is probable, because all *Anopheles* sample pools in the current work were ISV-positive.

Third, characterization of the arthropod virome may shed light on the evolution of mosquito antiviral immune mechanisms, as well as the evolution of pathogenic arboviruses. ISV replication is restricted to insect cells, but the potential of most mosquito-associated viruses for transmission to humans or other vertebrates is currently unknown, because few studies of host range and transmission have been done. Some viruses may have a host range restricted to only *Anopheles*. For example, *Anopheles* cypovirus and *Anopheles* C virus replicate and are maintained by vertical transmission in *An. coluzzii*, but were not able to infect *Ae. aegypti* in exposure experiments, and infected *Anopheles stephensi* only transiently [4]. Thus, *Anopheles* ISVs may display fine host restriction to genus or even to particular *Anopheles* species and not others.

It is likely that the main evolutionary pressure shaping mosquito antiviral mechanisms is their persistent exposure in nature to members of the natural virome, rather than the probably less frequent exposure to vertebrate-pathogenic arboviruses. Maintenance of bacterial microbiome commensals in the non-pathogenic commensal state requires active policing by basal host immunity [36]. By analogy, the maintenance of persistent ISVs as non-pathogenic may also result from a dialog with host immunity. Presumably, the same antiviral mechanisms used in basal maintenance of ISVs are also deployed against arboviruses when encountered, which are not novel to the vector because they are often in the same families as members of the insect virome [2]. Knowledge of the mechanisms that allow *Anopheles* to carry a natural RNA virome, but apparently reject arboviruses, may provide new tools to raise the barrier to arbovirus transmission by the more efficient *Aedes* and *Culex* vectors.

In addition to the canonical immune signaling pathways, piRNAs can be involved in antiviral protection, although this research is just beginning [22, 37]. One function of genomic piRNA clusters appears to be storage of a molecular archive of genomic threats such as transposable elements, linked to an effector mechanism to inactivate them. This is analogous to bacterial molecular memory mediated by the CRISPR/Cas system. We identified two candidate piRNA genes that appear to be downregulated upon ONNV infection in *An. coluzzii*. Involvement of piRNAs during viral infection has not been previously demonstrated in *Anopheles*. piRNA monitoring of the virome may be part of the normal basal management of ISVs to limit their pathogenicity if not controlled, and our current results suggest that piRNA dynamics may also be involved in host response to an arbovirus. Further work including specific piRNA silencing studies will be required to draw these connections.

## Conclusions

The current report shows that the *Anopheles* virome is complex and diverse, and can be influenced by the geography of mosquito species. This is exemplified by the fact that some viruses are restricted to *Anopheles* in Senegal, and others in Cambodia. Similar results were seen in *Ae. aegypti,* where five ISVs were specific to the Australian host population, while six others were found only in the Thai host population [38]. Differences in the *Anopheles* virome across geography could be explained by climate, environmental conditions, breeding sites, and mosquito bloodmeal sources, among other factors. The presence in this study of such a large number of novel and unclassified virus assemblies highlights the fact that the malaria vector virome is understudied. The same observation has been made during metagenomics surveys in *Drosophila*, *Aedes* and *Culex* [28, 39, 40] among other arthropods, indicating that the vast majority of insect viruses are not yet discovered.

## Methods

### Sample collections

Mosquitoes were collected in Cambodia in Kres village, Ratanakiri province (sample pools Cam5–02 and Cam10–02) and Cheav Rov village, Kampong Chhnang province (sample pools Cam5–01 and Cam10–01). The majority of inhabitants are engaged in forest-related activities (agriculture, logging and hunting) and may spend the night in forest plots during the harvest period. Vegetation varies from evergreen forest to scattered forest, and the dry season typically runs from November to May and the rainy season from June to October. In Senegal, sampling sites were located in the department of Kedougou in southeastern Senegal. Kedougou lies in a transition zone between dry tropical forest and the savanna belt, and includes the richest and most diverse fauna of Senegal. Recent arbovirus outbreaks include CHIKV in 2009–2010, yellow fever virus in 2011, Zika virus in 2010, and DENV in 2008–2009.

Permission to collect mosquitoes was obtained by Institut Pasteur Cambodia from authorities of Ratanakiri and Kampong Chhnang, and by Institut Pasteur Dakar from authorities of Kedougou. Wild mosquitoes visually identified as *Anopheles* spp. at the collection site (non-*Anopheles* were not retained) were immediately transferred into RNAlater stabilization reagent kept at 4 °C, and then returned to the laboratory and stored at − 80 °C until RNA extraction.

### RNA extraction, library construction, and sequencing

Total RNA was extracted from four pools of mosquitoes from each of Senegal and Cambodia (Senegal sample pools: 5 mosquitoes, Dak5–03, Dak5–04, 10 mosquitoes, Dak10–03, Dak10–04; Cambodia sample pools: 5 mosquitoes, Cam5–01, Cam5–02, 10 mosquitoes, Cam10–01, Cam10–02) using the Nucleospin RNA kit (Macherey-Nagel) following the supplied protocol. Library preparation and sequencing steps were performed by Fasteris (Plan-les-Ouates, Switzerland, www.fasteris.com). Long RNA libraries from the eight mosquito pools were made from total RNA depleted of ribosomal RNA by treatment with RiboZero (Illumina, San Diego, CA). Libraries were multiplexed and sequenced on a single lane of the Illumina HiSeq 2500 platform (Illumina, San Diego, CA) by the paired-ends method (2 × 125 bp), generating on average 36 million high-quality read pairs per library. Small RNA libraries with insert size 18–30 nt were generated from the same eight mosquito pools as above, multiplexed and sequenced in duplicate (two technical replicates per pool) in two lanes of the Illumina HiSeq2500 platform (Illumina, San Diego, CA) by the single-end method (1 × 50 bp) generating on average 34 million reads of high-quality small RNA reads per library.

### Pre-processing of long and small RNA libraries

Cutadapt 1.13 [41] was used for quality filtering and adaptor trimming of reads from long and small RNA libraries. Low-quality 3′ ends of long RNA reads were trimmed by fixing a phred quality score of 15, and reads smaller than 50 bp after quality filtering and adaptor trimming were removed. In the case of small RNA libraries, reads shorter than 15 bp after quality filtering and adaptor trimming were removed.

In order to filter sequences originating in the mosquito host, sequences passing the above quality filter step were mapped against a custom database consisting of 24 *Anopheles* genomes available in Vectorbase in February 2016 [42]. Bowtie 1.2.0 [43] was used to map small RNA libraries with two mismatches allowed, whereas the BWA-MEM algorithm from BWA-0.7.12 [44] with default parameters was used to map long RNA libraries. Sequence reads that did not map against *Anopheles* genomes, herein referred to as non-host processed reads, were retained and used for de novo assembly and subsequent binning of virus transcripts.

### Estimation of *Anopheles* species composition of mosquito sample pools

Quality-filtered long RNA read pairs were mapped with SortMeRNA [45] against a custom database of *Anopheles* sequences of the mitochondrial cytochrome c oxidase subunit 1 gene (COI-5P database) extracted from the Barcode of Life database [46]. 98% identity and 98% alignment coverage thresholds were fixed for the operational taxonomic unit calling step of SortMeRNA. Operational taxonomic unit counts were collapsed at species level and relative abundances of *Anopheles* species with at least 100 reads and 1% frequency in the sample pool were represented as pie charts using the ggplots2 R package.

### De novo sequence assembly and identification of virus contigs by sequence similarity

Processed reads from each country (Cambodia and Senegal) were combined and de novo assembled using different strategies for long and small RNA libraries. Small RNA reads were assembled using the Velvet/Oases pipeline [47] using a range of k-mer values from 13 to 35. Long RNA reads were assembled using both the Velvet/Oases pipeline with a range of k-mer values from 11 to 67 and Trinity [48].

Contigs produced by parallel assembly of Cambodia and Senegal processed reads were filtered in order to remove trans-self chimeric sequences using custom shell scripts, and the resulting contigs were merged with cd-hit-est [49] (95% nucleotide identity over 90% alignment length) in order to generate a final set of non-redundant contig sequences. Non-redundant contigs longer than 500 nucleotides were compared against the GenBank protein sequence reference database using BLASTX [50] with an e-value threshold of 1e-10, and the results were imported into MEGAN6 in order to classify contigs taxonomically using the LCA algorithm [51]. Contigs of viral origin were further subjected to manual curation by pairwise sequence alignments of nucleotide sequences using BLASTN, and of translated query sequences searched against the translated nucleotide database using TBLASTX and the Easyfig genome comparison tool [52] in order to remove redundancies not detected in previous steps. Sequence assemblies and annotations are available in Additional file 7: Classified Virus Sequences and Additional file 8: Unclassified Virus Sequences.

### Structural and functional annotation of virus assemblies

Assembled contigs of viral origin were annotated as follows: ORFs were predicted with MetaGeneMark [53], and functionally annotated using Prokka [54] with Virus kingdom as primary core reference database for initial BLASTP searches and including also as reference Hidden Markov Models of virus protein families defined in vFam database [55]. Also, protein sequences of predicted ORFs were processed with the Blast2GO pipeline [56], which generates functional annotation of proteins from BLASTP results against the virus subdivision of GenBank as well as Gene Ontology annotations from top BLASTP results. Prediction of InterPro signatures over viral proteins was also carried out with the InterProScan tool integrated in Blast2GO. The results of the different strategies of structural and functional annotation were integrated and manually curated with Artemis [57].

### Phylogenetic analyses

In order to place the new virus sequences characterized in the present study into an evolutionary context, the peptide sequences of RdRP ORFs detected in the annotation step were aligned with the corresponding homologs in reference positive-sense and negative-sense single-strand RNA viruses (ssRNA) and double strand RNA (dsRNA) viruses using MAFFT v7.055b with the E-INS-i algorithm [58]. Independent alignments were generated for all ssRNA and dsRNA viruses and for different virus families (Bunya-Arenavirus, Monenegavirus, Orthomyxovivirus, Flavivirus, Reovirus). The resulting alignments were trimmed with TrimAI [59] in order to remove highly variable positions, keeping the most conserved domains for phylogenetic reconstruction. Phylogenetic trees were reconstructed by maximum likelihood with RAxML [60] with the WAG+GAMMA model of amino acid substitution and 100 bootstrap replicates. Phylogenetic trees were visualized with the R package Ape [61].

### Prediction of unclassified contigs of viral origin by small RNA size profiling

In order to recruit contigs of potential viral origin from the pool of unclassified transcripts, we use the approach of Aguiar [19]. This approach uses the size profiles of small RNA reads that maps over positive and negative strands of viruses detected by sequence similarity as a signature to identify unclassified transcripts by sequence similarity of potential viral origin. For this purpose, processed small RNA reads were re-mapped over virus contigs and unclassified contigs by sequence similarity using bowtie 1.2.0 [43] allowing at most one mismatch and retaining only those contigs with at least 100 small RNA reads mapped. From the mapped small RNA reads over each contig, the small RNA size profiles were defined as the frequency of each small RNA read of size from 15 to 35 nucleotides that map over the positive and negative strand of the reference sequence. To compute these small RNA size profiles, reads mapped over positive and negative strands of each reference sequence were extracted with Samtools [62], and the size of small RNA reads were computed with the Infoseq program of the EMBOSS package [63]. Custom shell scripts were used to parse Infoseq output to a matrix representing the frequency of reads of different sizes and polarity across virus/unclassified contigs. This matrix was further processed in R (version 3.3.2). In order to normalize the small RNA size profiles, a z-score transformation is applied over the read frequencies of each contig (virus/unclassified). The similarity between small RNA size profiles of virus and unclassified contigs is computed as the Pearson correlation coefficient of the corresponding z-score profiles, and the relationship between small RNA size profiles of virus/unclassified contigs was defined from this similarity values using UPGMA as linkage criterion with the R package Phangorn [64]. These relationships were visualized as heatmaps of the z-score profiles in R with gplots package (version 3.0.1) using the UPGMA dendrogram as the clustering pattern of virus/unclassified sequences. Unclassified contigs with a Pearson correlation coefficient of at least 0.9 with virus contigs and coming from the same mosquito sample pool were regrouped into clusters.

### ONNV infection and candidate piRNA gene regulation

Infection of *An. coluzzii* with ONNV, library preparations, and sequencing were described [65]. Briefly, small RNA sequence reads from 2 biological replicate pools of 12 mosquitoes each fed an ONNV-infected bloodmeal (unfed mosquitoes removed), and 2 replicate control pools of 12 mosquitoes each fed an uninfected normal bloodmeal were mapped to the *An. gambiae* PEST AgamP4 genome assembly using STAR version 2.5 with default parameters [66]. The resulting SAM files were analyzed using the Cuffdiff function in Cufflinks version 2.2.1 to test for differential abundance of small RNAs mapping to candidate piRNA genes, as compared between ONNV infected and control uninfected samples. This analysis yielded 86 candidate piRNA genes that were differentially represented in the small RNA sequences between the ONNV and control treatment conditions (Additional file 6: Table S4). The candidate piRNA genes used were previously described in 187 genomic piRNA clusters, and are listed in the annotation file, GOL21-bonafide-piRNAs-24-29 nt.fastq (from [25], publicly available from Figshare at doi 10.6084/m9.figshare.7308518). The piRNAs of *An. coluzzii* were designated in [25] as either novel genes (denoted XLOC loci), and as piRNAs produced from within existing genes of the PEST genome assembly (denoted AGAP loci).

Independent confirmation of the Cuffdiff analysis was obtained using BAM and BAI indices generated using Bowtie 2 version 2.3.0 from the above small RNA sequence files of ONNV infected and uninfected samples. These generated files were analyzed with the *An. gambiae* PEST AgamP4 genome assembly in the Integrative Genomics Viewer version 2.5 [67]. We quantified the small RNA sequence reads mapping to the piRNA gene candidates, XLOC_012931 and XLOC_012762, identified as differentially expressed by the Cuffdiff analysis. Mapped reads to each piRNA candidate gene were normalized using the library size of each sequence. Graphpad Prism 7 was used to create graphs from normalized reads, and statistical tests were performed using R version 3.5.2 [68].

## Additional files

Additional file 1: **Table S1.** *Anopheles* mosquito taxa represented in the collections from Senegal and Cambodia, as detected by comparison to *Anopheles* sequences from the Barcode of Life COI-5P database. Data corresponds to pie charts of *Anopheles* taxa by country and sample pool depicted in Fig. 1. (XLSX 29 kb)

Additional file 2: **Table S2.** List of 115 assembled virus contigs displaying sequence similarity to known virus families. (XLSX 11 kb)

Additional file 3: **Table S3.** Predicted open reading frames, peptide annotation and functional status of 115 assembled virus contigs displaying sequence similarity to known virus families. (XLSX 56 kb)

Additional file 4: **Figure S1.** Small RNA size profiles (A) and coverage profiles (B) of 15 novel virus assemblies with classic RNAi processing pattern. Virus assemblies shown are in Fig. 5, Cluster 3, and belong to the 115 novel viruses classified by sequence similarity to known virus assemblies. Red vertical bars represent reads mapped over the positive strand of reference viral sequence, and blue bars represent reads mapped over the negative strand. (PDF 287 kb)

Additional file 5: **Figure S2.** Small RNA size profiles of high-quality assemblies left unclassified by sequence similarity grouping. Thirty-nine unclassified contigs that meet the same quality criteria as the 115 classified contigs (non-redundant and > 500 nucleotides), and with at least 100 small RNA sequence reads. The 39 contigs display strong association by small RNA profile Cluster 2 and Cluster 3 (Fig. 5), which are enriched for 21 nucleotide reads mapping over both positive and negative strands, characteristic of the classical siRNA product size profile. Red bars represent reads mapped over the positive strand of reference viral sequence, and blue bars represent reads mapped over the negative strand. (PDF 29 kb)

Additional file 6: **Table S4.** *Anopheles coluzzii* candidate piRNA genes differentially represented in small RNA sequence of ONNV infected and uninfected controls. (XLSX 18 kb)

Additional file 7: Classified Virus Sequences. Sequence assemblies and annotations for the final 115 novel virus contigs non-redundant contigs > 500 nucleotides, classified by sequence similarity to known viruses. In EMBL flat sequence file format. (ZIP 208 kb)

Additional file 8: Unclassified Virus Sequences. Sequence assemblies for 39 non-redundant contigs > 500 nucleotides, unclassified by sequence similarity to known viruses. The small RNA profile matched a pattern of classic RNAi processing of viral replication intermediates, suggesting they are undescribed viruses. In fasta text sequence file format. (FASTA 38 kb)

## Data Availability

All sequence files are available from the EBI European Nucleotide Archive database (http://www.ebi.ac.uk/ena/) under project accession PRJEB29993 (datasets ERR3275139-ERR3275154 for small RNA libraries; datasets ERR2939203- ERR2939210 for long RNA libraries). Sequence assemblies and annotations for the 115 novel virus contigs are available in Additional file 7: Classified Virus Sequences. Sequence assemblies of 39 unclassified viruses are available in Additional file 8: Unclassified Virus Sequences.

## Abbreviations

CHIKV: Chikungunya virus
COI: Cytochrome c oxidase subunit 1
d: Days
DENV: Dengue virus
dsRNA: Double strand RNA
ISV: Insect specific virus
ONNV: O’nyong nyong
ORF: Open reading frame
PCLV: Phasi Charoen-like virus
piRNA: Piwi-interacting RNA
RdRP: RNA-dependent RNA polymerase
RNAi: RNA interference
RPKM: Reads per kilobase per million reads
siRNA: Small interacting RNA
ssRNA: Single-stranded RNA
ZIKV: Zika virus
