# Correction to: De novo profiling of RNA viruses in *Anopheles* malaria vector mosquitoes from forest ecological zones in Senegal and Cambodia

**DOI:** 10.1186/s12864-019-6067-5

**Published:** 2019-09-05

**Authors:** Eugeni Belda, Ferdinand Nanfack-Minkeu, Karin Eiglmeier, Guillaume Carissimo, Inge Holm, Mawlouth Diallo, Diawo Diallo, Amélie Vantaux, Saorin Kim, Igor V. Sharakhov, Kenneth D. Vernick

**Affiliations:** 1 Unit of Insect Vector Genetics and Genomics, Department of Parasites and Insect Vectors, Institut Pasteur, Paris, France; 2 CNRS Unit of Evolutionary Genomics, Modeling, and Health (UMR2000), Institut Pasteur, Paris, France; 3 Integromics Unit, Institute of Cardiometabolism and Nutrition, Assistance Publique Hôpitaux de Paris, Pitié-Salpêtrière Hospital, Paris, France; 4 Sorbonne Université, Graduate School of Life Sciences ED515, UPMC - Université Pierre et Marie Curie - Paris 6, 4 Place Jussieu, 75252 Paris, France; 5 Laboratory of Microbial Immunity, Singapore Immunology Network, Agency for Science, Technology and Research (A(∗)STAR), Singapore, Singapore; 6 Institut Pasteur de Dakar, Dakar, Senegal; 7 Institut Pasteur of Cambodia, Phnom Penh, Cambodia; 8 Department of Entomology, Virginia Polytechnic Institute and State University, Blacksburg, VA USA

**Correction to: BMC Genomics**

**https://doi.org/10.1186/s12864-019-6034-1**

Following the publication of this article [1], the authors reported that the original shading in columns 3 and 4 of Table 3, which indicated the presence or absence of viruses in each library, had been removed during typesetting.

A corrected Table 3 indicating the presence or absence of viruses is included in this Correction article. The publisher would like to apologize to the authors and any readers for the inconvenience.

**Table 3:** Similarity of Senegal and Cambodia virus assemblies by BLASTX to 24 reference viruses in GenBank. 10 targets are shared, 9 are Senegal-specific, and 5 are Cambodia-specific

Reference virus	Viral taxonomy	Senegal libraries	Cambodia libraries
Culex tritaeniorhynchus rhabdovirus RNA, complete genome	Viruses; ssRNA viruses; ssRNA negative-strand viruses; Mononegavirales; Rhabdoviridae; unclassified Rhabdoviridae.	Present	Present
Phasi Charoen-like virus	Viruses; ssRNA viruses; ssRNA negative-strand viruses; Bunyavirales; unclassified Bunyavirales.	Present	Present
Uncultured virus isolate acc_1.3	Viruses; environmental samples.	Present	Present
Wellfleet Bay virus isolate 10–280-G segment 4	Viruses; ssRNA viruses; ssRNA negative-strand viruses; Orthomyxoviridae; Quaranjavirus; unclassified Quaranjavirus.	Present	Present
Wuhan Mosquito Virus 1 strain WT3–15	Viruses; ssRNA viruses; ssRNA negative-strand viruses; unclassified ssRNA negative-strand viruses.	Present	Present
Wuhan Mosquito Virus 9 strain JX1–13	Viruses; ssRNA viruses; ssRNA negative-strand viruses; unclassified ssRNA negative-strand viruses.	Present	Present
Xincheng Mosquito Virus strain XC1–6	Viruses; ssRNA viruses; ssRNA negative-strand viruses; unclassified ssRNA negative-strand viruses.	Present	Present
Xinzhou Mosquito Virus strain XC3–5	Viruses; ssRNA viruses; ssRNA negative-strand viruses; unclassified ssRNA negative-strand viruses.	Present	Present
Beaumont virus strain 6	Viruses; ssRNA viruses; ssRNA negative-strand viruses; Mononegavirales; Rhabdoviridae; unclassified Rhabdoviridae.	Present	Present
Jurona virus	Viruses; ssRNA viruses; ssRNA negative-strand viruses; Mononegavirales; Rhabdoviridae; Vesiculovirus.	Present	Present
Omono River virus	Viruses; dsRNA viruses	Present	Absent
American dog tick phlebovirus isolate FI3	Viruses; ssRNA viruses; ssRNA negative-strand viruses; Bunyavirales; Phlebovirus; unclassified Phlebovirus.	Present	Absent
Daeseongdong virus 1 strain A12.2708/ROK/2012	Viruses; unclassified viruses.	Present	Absent
DsRNA virus environmental sample clone mill.culi_contig84	Viruses; dsRNA viruses; environmental samples.	Present	Absent
Homalodisca vitripennis reovirus segment S3	Viruses; dsRNA viruses; Reoviridae; Sedoreovirinae; Phytoreovirus; unclassified Phytoreovirus	Present	Absent
Ixodes scapularis associated virus 2 isolate A1, partial genome	Viruses; unclassified viruses.	Present	Absent
Sunn-hemp mosaic virus	Viruses; ssRNA viruses; ssRNA positive-strand viruses, no DNA stage; Virgaviridae; Tobamovirus.	Present	Absent
Uncultured virus isolate acc_7.4	Viruses; environmental samples.	Present	Absent
Wuhan Spider Virus strain SYZZ-2	Viruses; ssRNA viruses; ssRNA negative-strand viruses; unclassified ssRNA negative-strand viruses.	Present	Absent
Nienokoue virus isolate B51/CI/2004	Viruses; ssRNA viruses; ssRNA positive-strand viruses, no DNA stage; Flaviviridae	Absent	Present
Oat golden stripe virus RNA1	Viruses; ssRNA viruses; ssRNA positive-strand viruses, no DNA stage; Virgaviridae; Furovirus	Absent	Present
Puerto Almendras virus isolate LO-39	Viruses; ssRNA viruses; ssRNA negative-strand viruses; Mononegavirales; Rhabdoviridae; unclassified Rhabdoviridae.	Absent	Present
Tobacco streak virus isolate pumpkin segment RNA1	Viruses; ssRNA viruses; ssRNA positive-strand viruses, no DNA stage; Bromoviridae; Ilarvirus	Absent	Present
Bivens Arm virus isolate UF 10	Viruses; ssRNA viruses; ssRNA negative-strand viruses; Mononegavirales; Rhabdoviridae; Tibrovirus	Absent	Present
