# Supplementary figures and images for: De novo profiling of RNA viruses in Anopheles malaria vector mosquitoes from forest ecological zones in Senegal and Cambodia
Source: BMC Genomics. 2019 Aug 20;20:664. doi: 10.1186/s12864-019-6034-1 (PMC6702732; doi:10.1186/s12864-019-6034-1)

**A**

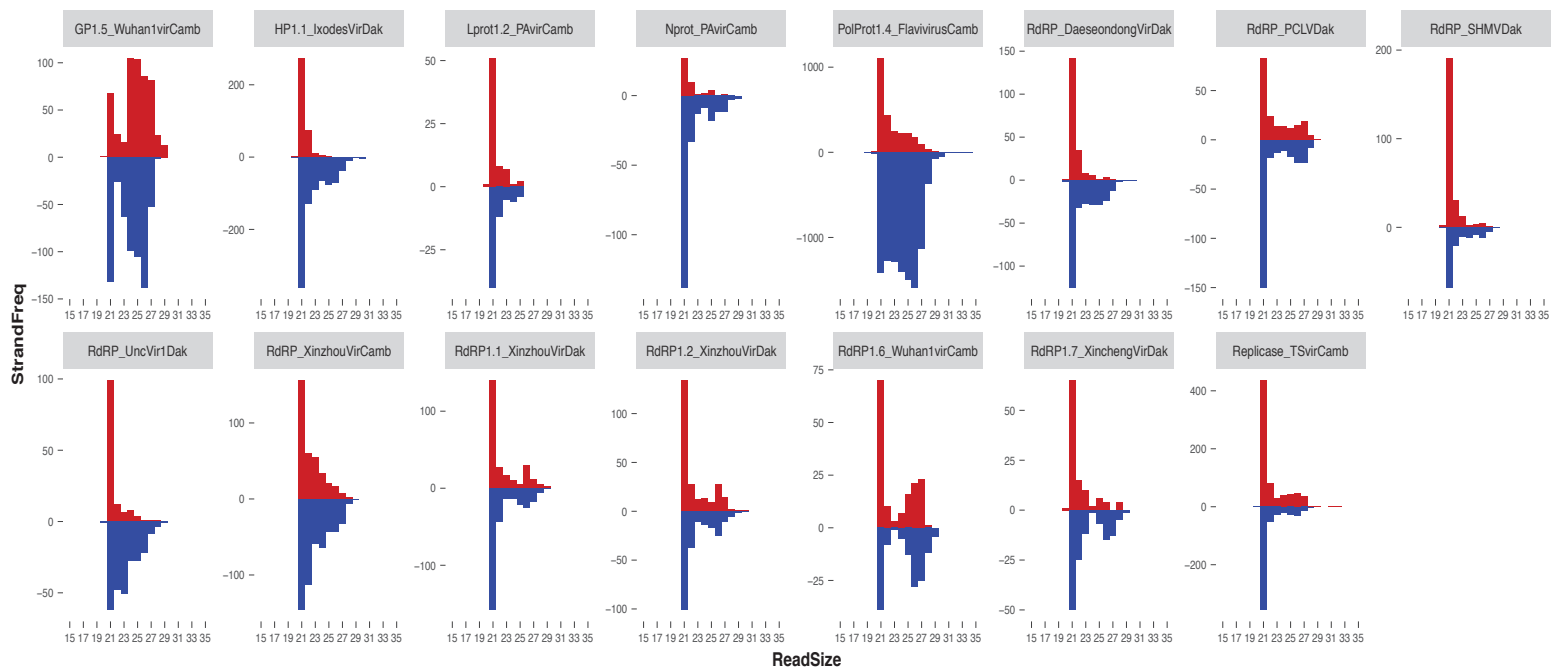

**B**

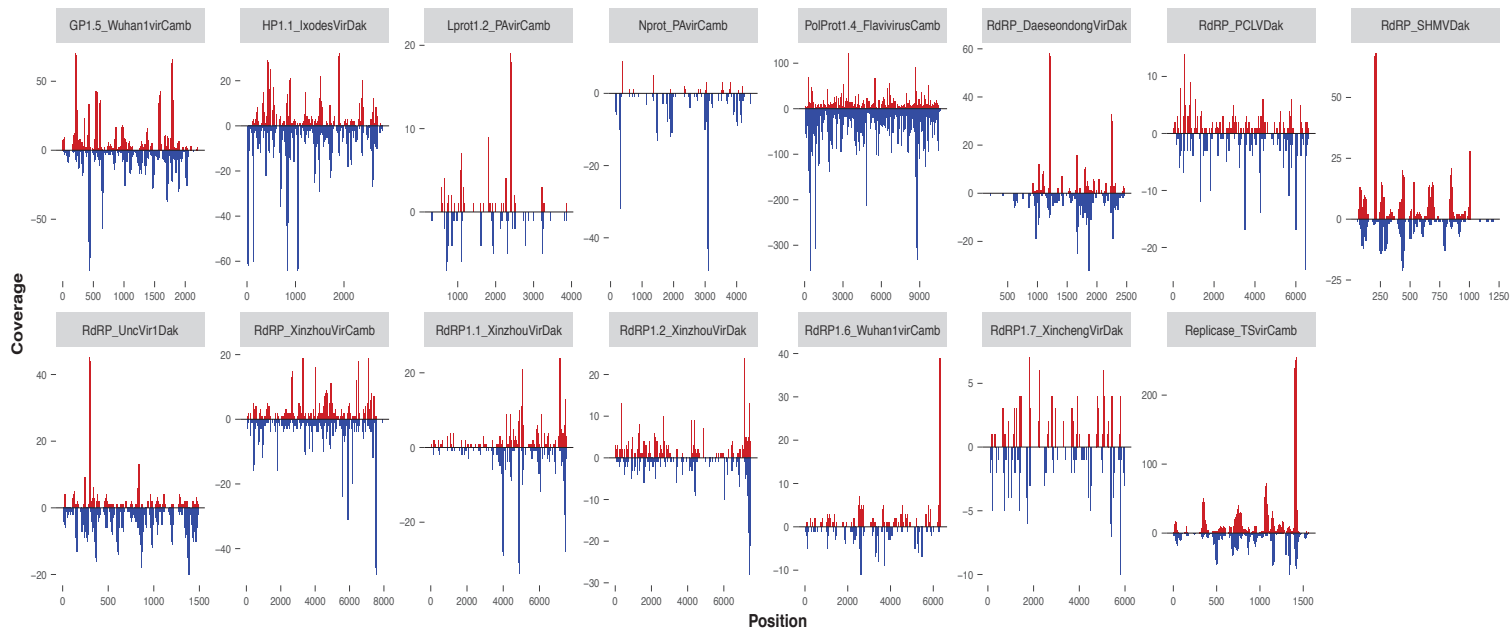

Supplement: Supplementary file 4 — Figure S1. Small RNA size profiles (A) and coverage profiles (B) of 15 novel virus assemblies with classic RNAi processing pattern. Virus assemblies shown are in Fig. 5, Cluster 3, and belong to the 115 novel viruses classified by sequence similarity to known virus assemblies. Red vertical bars represent reads mapped over the positive strand of reference viral sequence, and blue bars represent reads mapped over the negative strand. (PDF 287 kb) [file 12864_2019_6034_MOESM4_ESM.pdf]

StrandFreq

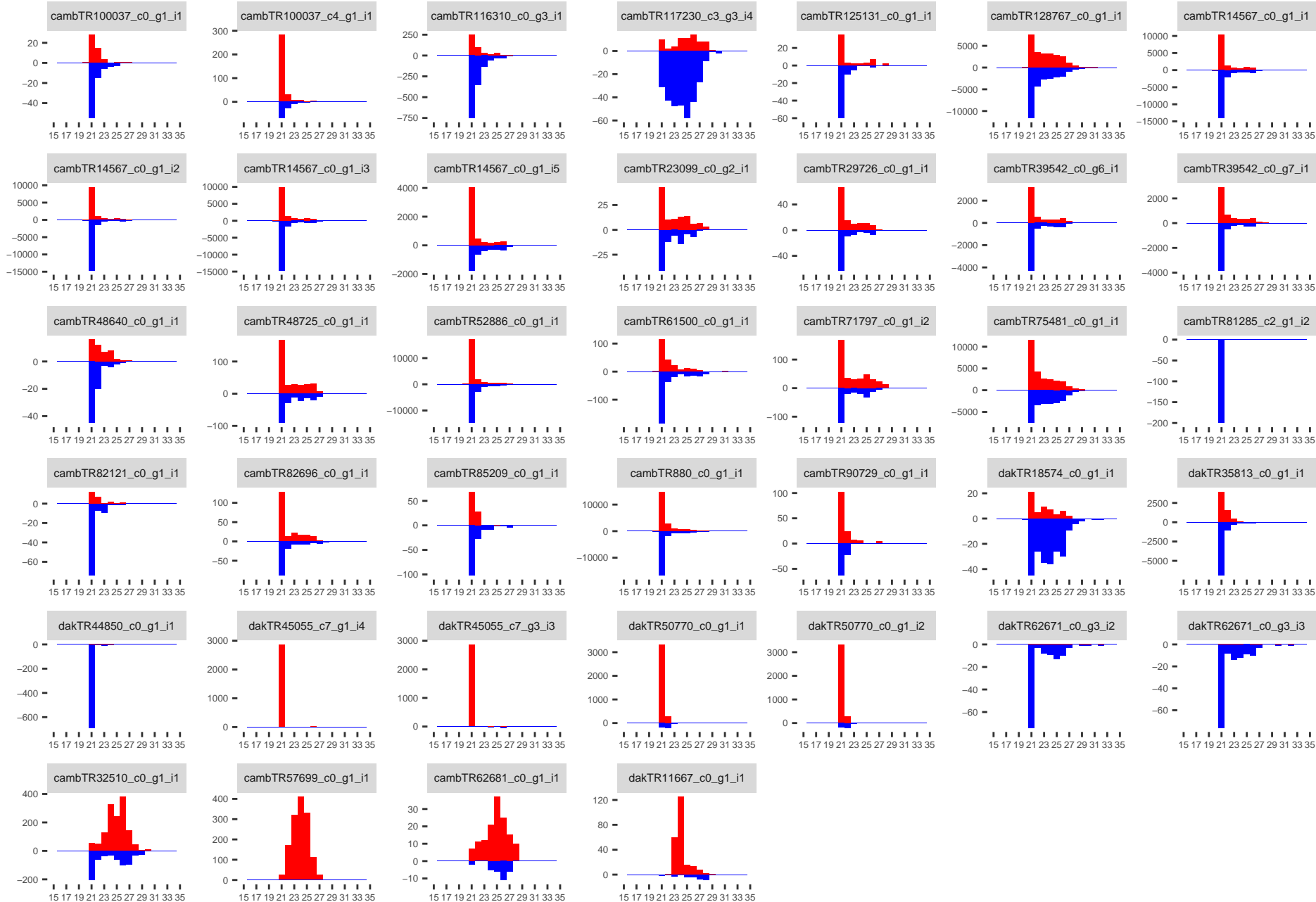

ReadSize

Supplement: Supplementary file 5 — Figure S2. Small RNA size profiles of high-quality assemblies left unclassified by sequence similarity grouping. Thirty-nine unclassified contigs that meet the same quality criteria as the 115 classified contigs (non-redundant and > 500 nucleotides), and with at least 100 small RNA sequence reads. The 39 contigs display strong association by small RNA profile Cluster 2 and Cluster 3 (Fig. 5), which are enriched for 21 nucleotide reads mapping over both positive and negative strands, characteristic of the classical siRNA product size profile. Red bars represent reads mapped over the positive strand of reference viral sequence, and blue bars represent reads mapped over the negative strand. (PDF 29 kb) [file 12864_2019_6034_MOESM5_ESM.pdf]
